# Every Trait Counts: Marginal Maximum Likelihood Estimation for Novel Multidimensional Count Data Item Response Models with Rotation or 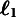 –Regularization for Simple Structure

**DOI:** 10.1017/psy.2024.17

**Published:** 2025-01-03

**Authors:** Marie Beisemann, Heinz Holling, Philipp Doebler

**Affiliations:** 1 Department of Statistics, TU Dortmund University, Dortmund, Germany; 2 Institute of Psychology, University of Münster, Münster, Germany

**Keywords:** 2PCMPM, Conway–Maxwell–Poisson distribution, count data, EM algorithm, item response theory, lasso regularization, multidimensional IRT

## Abstract

Multidimensional item response theory (MIRT) offers psychometric models for various data settings, most popularly for dichotomous and polytomous data. Less attention has been devoted to count responses. A recent growth in interest in count item response models (CIRM)—perhaps sparked by increased occurrence of psychometric count data, e.g., in the form of process data, clinical symptom frequency, number of ideas or errors in cognitive ability assessment—has focused on unidimensional models. Some recent unidimensional CIRMs rely on the Conway–Maxwell–Poisson distribution as the conditional response distribution which allows conditionally over-, under-, and equidispersed responses. In this article, we generalize to the multidimensional case, introducing the Multidimensional Two-Parameter Conway–Maxwell–Poisson Model (M2PCMPM). Using the expectation-maximization (EM) algorithm, we develop marginal maximum likelihood estimation methods, primarily for exploratory M2PCMPMs. The resulting discrimination matrices are rotationally indeterminate. Recently, regularization of the discrimination matrix has been used to obtain a simple structure (i.e., a sparse solution) for dichotomous and polytomous data. For count data, we also (1) rotate or (2) regularize the discrimination matrix. We develop an EM algorithm with lasso ( 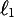 ) regularization for the M2PCMPM and compare (1) and (2) in a simulation study. We illustrate the proposed model with an empirical example using intelligence test data.

Multidimensional item response theory (MIRT) provides a framework in which responses to a set of items are explained by the items’ relation to a number of latent traits (Reckase, 2009). We assume that person *i*’s response to item *j* is influenced by *L* latent traits 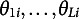 , where the influence strength is determined by discrimination parameters 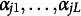 similar to factor loadings in linear factor analysis. The discrimination parameters for all items and all traits are contained in the discrimination matrix 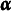 . The assumption of a number of latent traits—rather than just one, as in more traditional unidimensional item response models—is often considered more realistic in psychological research. Psychological constructs are often by definition composed of multiple subcomponents, or response behavior is assumed to be complex and multifactorial.

Multidimensional item response models can be divided into confirmatory and exploratory models, analogous to the factor analytical tradition (McDonald, 1999). While confirmatory models test the fit of a prespecified item–trait relationship structure to the data, exploratory models aim to determine which items stand in relation to which factors, for instance through rotation of the discrimination (or factor loadings) matrix 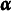 . A common goal of this popular method is to find a simple structure, that is, an item–trait relationship structure where each item loads primarily onto one factor and not (or only to a small extent) on the remaining factors (Browne, 2001; Thurstone, 1947). An alternative strategy to this end—which has only recently gained popularity in the context of MIRT—is regularization (Cho et al., 2022; Sun et al., 2016). Regularization includes techniques often originally developed for variable selection in (generalized) linear models (Hastie et al., 2009). By including a penalty term in the model likelihood, sparse parameter estimates with many zeroes can be enforced. In comparison to unpenalized estimation, parameter values are shrunken toward 0, often improving predictive performance and model interpretation. In the context of MIRT, this leads to more parsimonious estimates of discrimination matrices 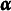 by selecting only notable item-trait relationships and shrinking the rest toward 0 (see also Trendafilov, 2014).

Research into regularization as a tool to find simply structured discrimination matrices 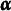 in MIRT models has so far focused on models for binary and ordinal response data. But some psychometric tests and self-reports generate another type of response data: counts. For instance, divergent thinking and verbal fluency tasks (Myszkowski & Storme, 2021), or processing speed tasks (e.g., Doebler & Holling, 2016). Psychological count responses also occur among self-reports (e.g., in clinical psychology; Magnus & Thissen, 2017; Wang, 2010), or as biometric indicators (e.g., number of fixations in eye-tracking; Man & Harring, 2019). Count data naturally occur in text data analysis (Proksch & Slapin, 2009). Corresponding count data item response models have received increasingly more attention in the psychometric literature in recent years (e.g., Beisemann, 2022; Forthmann et al., 2020; Graßhoff et al., 2020; Man & Harring, 2019).

The simplest count data item response model, Rasch’s Poisson Counts Model (RPCM; Rasch, 1960; see also, e.g., Holling et al., 2015; Jansen, 1995; Jansen & van Duijn, 1992), models the expected count response 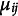 for person *i* to item *j* as 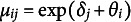 , where 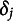 is the item easiness and 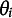 is the sole latent trait.^1^ Conditional (upon 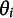 ) responses are assumed to follow a Poisson distribution. Extensions of the RPCM provided more general models, for example, by substituting the log-linear relationship in the RPCM by a sigmoid curve (Doebler et al., 2014), or by addressing the conditional equidispersion implied by the Poisson distribution. Conditional equidispersion leads to the strong assumption that 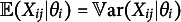 . Early extensions of the RPCM allowed overdispersed (i.e., 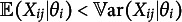 ) conditional response distributions (e.g., Hung, 2012; Wang, 2010). More recently, models for item-specific conditional equi-, over-, or underdispersion (i.e., 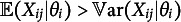 ) were proposed by employing the more general Conway–Maxwell–Poisson (CMP) distribution (Conway & Maxwell, 1962; Huang, 2017; Shmueli et al., 2005). The Conway Maxwell Poisson Model (CMPCM; Forthmann et al., 2020) has no discrimination parameters like a Rasch model, while the Two Parameter Conway Maxwell Poisson Model (2PCMPCM; Beisemann, 2022) includes discrimination parameters. Qiao et al. (2023) propose a CMP-based joint modeling approach. Tutz (2022) provides an alternative approach all together for dispersion handling. Regardless of the approach, the adequate consideration of dispersion for count data is important to ensure proper uncertainty quantification, i.e., correct standard errors and model-implied reliability (Forthmann et al., 2020).

These generalizations have focused on unidimensional count item response models. Apart from bidimensional extensions of RPCM (Forthmann et al., 2018, for a model without discrimination parameters, and Myszkowski & Storme, 2021, for a two-parameter Poisson model), multidimensional count data models have mostly been developed within the frameworks of generalized linear latent and mixed models (GLLAMM; Skrondal & Rabe-Hesketh, 2004) or factor analysis (Wedel et al., 2003) rather than within MIRT. These works have primarily relied on the Poisson distribution, with Wedel et al. (2003) accomodating some flexibility through truncation of the Poisson distribution leading to underdispersion, and allowing different link functions.

With the present work, we aim to generalize the 2PCMPM (Beisemann, 2022) to a multidimensional count data item response model framework which offers the advantages of multidimensional item response modeling for count data in conjunction with the dispersion flexibility of the CMP distribution. The framework contains a number of existing count data item response models as special cases, allowing for easy testing of assumptions by means of model comparisons. Our goal is further to provide marginal maximum likelihood estimation methods for the framework, with a focus on exploratory models. For these, interpretability of the discrimination matrix 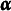 is a crucial goal and is aided by pursuing a simple structure for 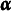 . To this end, we explore both traditional rotation techniques (Browne, 2001), and more novel regularization approaches (Hastie et al., 2009). The remainder of the paper is structured as follows: In the next section, we introduce and formulate the proposed multidimensional count data item response model framework. We proceed to present marginal maximum likelihood estimation methods for the framework, based on the expectation-maximization (EM) algorithm (Dempster et al., 1977). We present both unpenalized and penalized estimation methods. Afterward, we assess the proposed models and algorithms in a simulation study and illustrate the framework with a real-world application example. Finally, a discussion of the presented work is provided.

## 1 Multidimensional Two-Parameter Conway–Maxwell–Poisson models

The tests and self-reports for which methods are developed in this article consist of count data items. Item scores are calculated by counting events or by aggregating across a large number of tasks each with a binary score. From each participant 
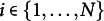
 , we obtain a response 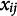 to each item 
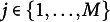
 , where 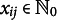 , 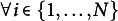 , 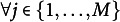 . An example of such count data tests in the psychological literature are tests in the creative thinking literature which ask participants for different associations in response to items (e.g., the alternate uses task, AUT, to assess divergent thinking; see e.g., Forthmann et al., 2020; Myszkowski & Storme, 2021 for psychometric analyses of AUT items). The associations given by each person *i* to each item *j* can be counted, resulting in the count response 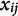 .

To model these count responses in an item response theory framework, we assume that the responses depend on item characteristics and *L* different latent traits 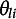 for person *i* and trait 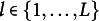 . When 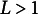 , the model is multidimensional. This assumption grants more flexibility as (1) unidimensional models are contained as special cases (for 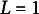 ) and (2) the assumption of more than one latent trait is often frequently more realistic and is often empirically supported. An overarching latent trait can be made up of different subdomains that influence item responses differently. Items may also share commonalities beyond the main unidimensional trait they measure, violating the local independence assumption in unidimensional models (in the AUT example, this could be different domains the items tap into like figural or verbal; Forthmann et al., 2018; Myszkowski & Storme, 2021). In a multidimensional framework, this can be accounted for by modeling the item domains as different latent traits.

We propose to extend the recently proposed two-parameter Conway–Maxwell–Poisson model (2PCMPM; Beisemann, 2022)—which models differing item discriminations and dispersions in a unidimensional model—to the multidimensional case. The proposed Multidimensional Two-Parameter Conway–Maxwell–Poisson Models (M2PCMPM) assumes a log-linear factor model for the expected count response: 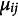


(1)

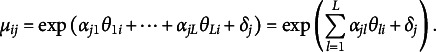

In this extension of the slope-intercept parametrized 2PCMPM, we denote by 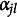 the slope for item *j* and trait *l*, which quantifies the extent to which differences in the latent trait *l* are reflected in the expected responses to item *j*. The parameter 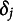 is the intercept for item *j*, which is related to—but does not directly correspond to—item *j*’s easiness. Analogously to the 2PCMPM, we then assume that responses follow a Conway–Maxwell–Poisson (CMP) distribution conditional on the *L* latent traits. We use the mean parameterization of the CMP distribution (Huang, 2017), denoted as 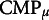 . Thus, we assume that (2)

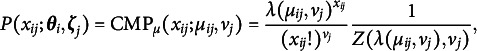

with 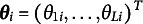 denoting the *L* latent traits of person *i*, 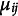 as in Equation (1) and 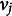 as the item-specific dispersion parameter ( 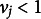 implies overdispersed, 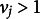 underdispersed, and 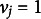 equidispersed conditional responses). In Equation (2), the expression 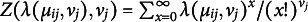 is the normalizing constant of the 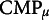 distribution (Huang, 2017). For simpler notation, we denote all item parameters 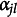 , 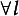 , 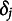 , and 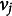 , for one item *j* concatenated in one vector with 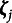 . As Huang (2017) showed, we obtain the rate 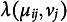 by solving (3)

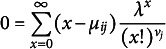

for 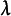 .

With the assumption of conditional independence given all *L* latent traits, the probability of the response vector 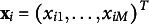 of person *i* is the product of Equation (2) for each item. The *L* latent traits 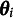 for each person *i* jointly follow a multivariate normal distribution with mean vector 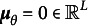 and covariance matrix 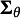 , where 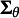 is a full rank 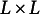 matrix with all diagonal entries equal to 1 for model identification purposes (more details on assumptions for 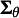 follow in section *Latent Trait Covariance Matrix*). Assuming that persons respond independently of each other, we obtain (4)

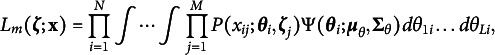

as the marginal likelihood for the data 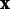 of all *N* respondents, where 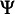 denotes the density of the multivariate normal distribution and 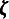 denotes the item parameters 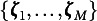 for all *M* items. The M2PCMPM contains a number of count data item response models as special cases which we list in Appendix C. In the following, we focus on exploratory M2PCMPMs, but see Appendix C for a note on confirmatory M2PCMPM within this framework.

### 1.1 Model identification

The full M2PCMPM as presented in Equation (1) constitutes an exploratory multidimensional item response model: Any item can be associated by any degree with any latent trait. For this reason, the full M2PCMPM as in Equation (1) is not uniquely identified; it is rotationally indeterminate. To enable estimation, we thus need to impose identification constraints on the discrimination matrix 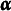 . A common constraint is a triangular 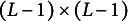 submatrix of zeroes in the discrimination matrix (as we believe is, for example, implemented in the mirt package; Chalmers, 2012), i.e., we impose constraints to 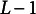 out of the *M* items to fix rotational indeterminacy. W.l.o.g., let these be the first 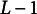 items. 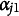 on the first trait is estimated freely and 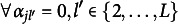 . For the following items 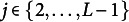 , the first *j* discriminations are free and we constrain 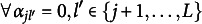 . In the following, this constraint will be referred to as the upper-triangle identification constraint. See, e.g., Sun et al., 2016, for examples, of alternative constraints. Note that imposing too strong or empirically insensible constraints may impact the model fit (negatively) (Sun et al., 2016). Identification constraints are imposed upon initial estimation to enable finding a likelihood mode. When rotating the initial solution, constraints are lifted, and the discrimination matrix 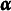 is rotated freely.

## 2 Marginal maximum likelihood estimation methods for the M2PCMPM

The goal of (frequentist) model estimation of the M2PCMPM is to maximize the model’s marginal likelihood (Equation (4)) in terms of item parameters 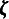 . An elegant and popular approach to marginal likelihood estimation in the context of item response models is the EM algorithm (Dempster et al., 1977; for an introduction see McLachlan & Krishnan, 2007; see Bock & Aitkin, 1981 for the first IRT application). The expected complete-data likelihood—rather than the observed marginal likelihood—is determined in each expectation (E) step. It includes unobservable parameters, i.e., the latent traits. The expected complete-data likelihood is maximized in each maximization (M) step. E and M steps are repeated until a convergence criterion is met.

### 2.1 Expectation-maximization algorithm

As the M2PCMPM is an extension of the 2PCMPM, estimation methods for the 2PCMPM can be extended to develop estimation methods for the M2PCMPM. Beisemann (2022) provided an EM algorithm for the 2PCMPM which we use as the basis for proposing EM algorithms for the M2PCMPM. The integral in Equation (4) does not exist in closed form and thus has to be approximated in estimation, for example, by Gauss–Hermite quadrature with fixed quadrature points. Relying on such a Gauss–Hermite quadrature for the integral approximation with 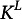 quadrature points, we generalize the expected complete-data log likelihood of the 2PCMPM (Beisemann, 2022) to 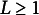 latent traits for the expected complete-data log likelihood of the M2PCMPM: (5)

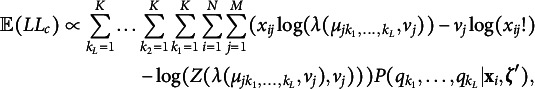

where 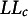 denotes the complete-data log likelihood, and (6)

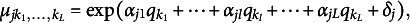

with 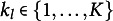 as the node index for trait *l*. Here, the joint posterior probability of the multidimensional quadrature point 
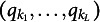
 is given by (7)

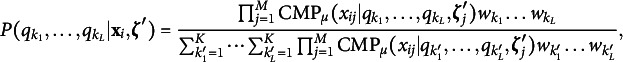

where with 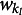 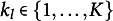 denote the nodes’ quadrature weights. The E step consists of computing Equation (7). In the subsequent M step, we maximize Equation (5) iteratively as a function of the item parameters 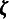 . To this end, we need to take the derivatives of Equation (5) with respect to the item parameters. We optimize in 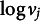 rather than 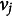 to search on an unconstrained parameter space (compare Beisemann, 2022). Similar to the techniques in Beisemann (2022) and Huang (2017), we form derivatives (using some results from Huang, 2017), resulting in gradients (8)

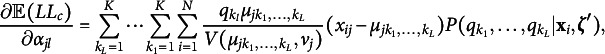

for slopes 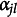 (note that 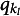 in the numerator of the fraction does not loop over all trait dimensions 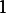 to *L*, but instead is specific to dimension 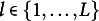 for the slope 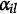 we are considering), (9)

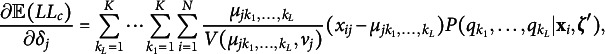

for intercepts 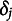 , and (10)

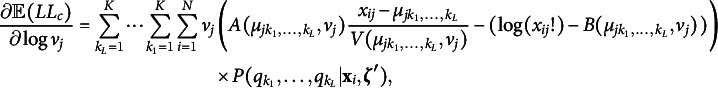

for log dispersions 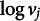 , with 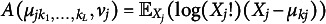 and 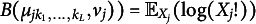 (Huang, 2017). Furthermore, (11)

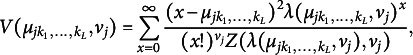

(Huang, 2017) is the variance of the 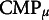 distribution which depends on 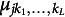 and 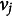 .

A known limitation of quadrature is its poor scaling to high dimensions (McLachlan & Krishnan, 2007); that is, in the context of the M2PCMPM, settings with greater numbers of latent traits. However, as illustrated with our example, in count data item response settings a smaller number of latent traits is frequently realistic.

### 2.2 Simple structure via rotation

After obtaining an initial solution with the EM algorithm described above, the classical approach for interpretable results is to apply a rotation to the discrimination parameters. Lifting the identification constraints after the initial solution is obtained, we have an infinite number of alternative solutions that can be obtained via rotation (i.e., rotational indeterminancy) (Scharf & Nestler, 2019). That is, there is an infinite number of rotation matrices 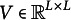 for which 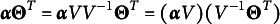 , where 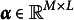 is the discrimination matrix and 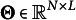 the latent trait matrix (Scharf & Nestler, 2019; Trendafilov, 2014). A preferred rotation matrix *V* has to be selected, usually one optimizing a specific criterion such as indicating a simple structure (Browne, 2001; Thurstone, 1947) of 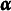 (Scharf & Nestler, 2019). Rotation techniques differ in the employed criterion and in whether they allow latent traits to be correlated (i.e., oblique methods) or not (i.e., orthogonal methods) (Scharf & Nestler, 2019; Trendafilov, 2014). Popular rotation techniques are for instance varimax (Kaiser, 1958, 1959), which is an orthogonal rotation method, and oblimin (Carroll, 1957; Clarkson & Jennrich, 1988), which is an oblique rotation method.

### 2.3 Simple structure via regularization

Recently, a simple structure has also been obtained with regularization techniques (Cho et al., 2022; Sun et al., 2016; Trendafilov, 2014). A perfect simple structure is a sparse matrix: Each item loads on exactly one latent trait, and the other loadings are zero (Scharf & Nestler, 2019; Trendafilov, 2014). Finding a sparse solution to an optimization problem is one aim of regularization (Hastie et al., 2009). By imposing a penalty term *R* onto the likelihood, regularization methods shrink parameter estimates toward 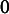 (Hastie et al., 2009). *R* is a function of all parameters to be regularized and grows as the absolute value of each parameter estimate grows (Scharf & Nestler, 2019). As a result, only substantial parameters (in our case, loadings or discriminations) remain notably different from 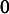 , essentially encouraging a (more) simple structure of the discrimination matrix 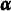 (Scharf & Nestler, 2019). As opposed to rotation methods, which are implemented after finding an initial estimate with the M2PCMPM EM algorithm, regularization methods modify the likelihood and have to be integrated into the EM algorithm. In general, the regularized estimates cannot be rotated without changing the value of *R*; they are hence rotationally determined in this sense.

As we maximize the expected complete-data log likelihood in each M step, we subtract the penalty term 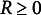 from it, weighted with a hyperparameter 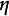 (notation here inspired by Scharf & Nestler, 2019; Sun et al., 2016 and in line with Beisemann, 2022). The penalty term *R* is a function of all slopes 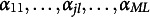 , as contained in 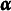 . We aim for a sparse solution specifically for 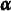 (ideally a simple structure), which is why we only impose the penalty term over 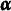 . We obtain (12)

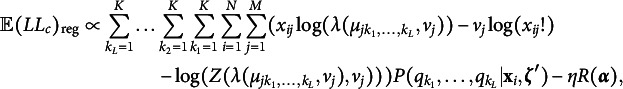

with 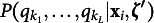 as in Equation (7). We can immediately see that for 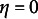 , the unregularized maximum likelihood estimate is optimal. The hyperparameter 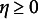 should be tuned, i.e., selected from a grid of possible values to provide the best result in terms of a tuning criterion (Hastie et al., 2009). We are going to return to this point further below.

Depending on the penalty term *R*, different regularization methods are implemented (for an introduction and an overview, see Hastie et al., 2009). In this work, we employ the lasso (Tibshirani, 1996) penalty, (13)

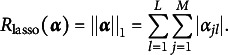

For binary and polytomous MIRT models, the lasso penalty has yielded promising results as a method to find a well-fitting discrimination matrix 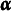 with a (rather) simple structure (Cho et al., 2022; Sun et al., 2016).

#### 2.4 Lasso penalty

Integrating the lasso penalty (Tibshirani, 1996) into the M2PCMPM EM algorithm requires an extension of the algorithm. We plug Equation (13) into Equation (12), and we observe that the E step of the M2PCMPM algorithm remains unaltered by the penalty term. In the M step, we are confronted with the problem that due to the 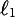 norm, the gradient only exists for 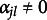 . To solve this issue for binary and polytomous MIRT models, Sun et al. (2016) employed the coordinate descent algorithm (Friedman et al., 2010) in the M step (see also Cho et al., 2022, for a related approach using variational estimation). Binary and polytomous MIRT models have an estimation advantage over count MIRT models in that they require only the estimation of discrimination and location parameters since the conditional variance is implied by these. The M2PCMPM additionally requires estimation of the dispersion parameters. A strategy in the context of (generalized) linear mixed models optimizing penalized (fixed) effects in one step, and then optimizing remaining model parameters in another step, alternating the steps until convergence (note that random effects are estimated in yet another step, but this is not of interest to us here; Nestler & Humberg, 2022; Schelldorfer et al., 2014). Inspired by these approaches, we propose the M2PCMPM lasso-EM algorithm (see Algorithm 1) that—during each M step—first optimizes 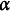 ’s and 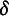 ’s using item-blockwise coordinate descent, and then optimizes dispersion parameters using Equation (10).

Taking an item-blockwise optimization approach as in Sun et al. (2016), we exploit that the expected complete-data log likelihood decomposes into the sum of the item contributions (immediately observable in Equation (5)). During each M step of the EM algorithm, we further assume (as is common in EM algorithms) the posterior probabilities from the previous E step for latent traits to be known (via the quadrature approximation). Thus, the (penalized) optimization problem during each M step and for each item *j* is that of a generalized linear model (GLM) with intercept 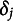 and (penalized) slopes 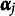 . Note that CMP 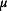 -regression is a “bona fide GLM[…]” (Huang, 2017, p. 365). This allows the use of algorithmic techniques developed for 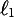 -regularization in GLMs, such as coordinate descent (Friedman et al., 2010).

As we can see in Algorithm 1 in Appendix B, we need updating rules for 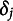 and the 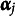 within the blockwise coordinate descent during the M step. To this end, we follow Sun et al. (2016): They approximate the expected complete-data log likelihood for item *j* (i.e., item-specific increment in Equation (5) in our case) as a univariate function of each item parameter, respectively, with a local quadratic approximation. Using this approximation, the resulting lasso update (with tuning parameter 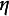 ) takes the following shape (Sun et al., 2016; adapted to our model and parameterization): (14)

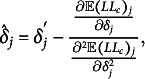

(Sun et al., 2016) for each 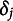 and (15)

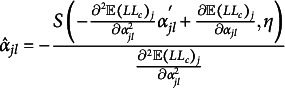

(Sun et al., 2016) for each 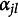 .^2^ Here, *S* denotes the soft thresholding operator (Donoho & Johnstone, 1995), which is defined as (16)

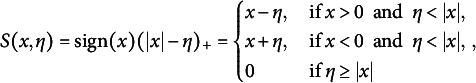

(Sun et al., 2016). We substitute the M2PCMPM-specific terms. 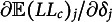 and 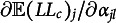 are given in Equations (8) and (9). Using the second derivatives of the variance 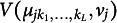 in terms of 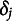 and 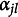 (see Appendix A) and results from Huang (2017), we obtain the following second derivatives in terms of 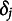 and 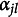 , (17)

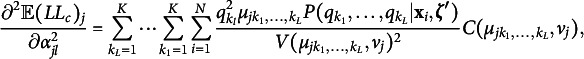

and (18)

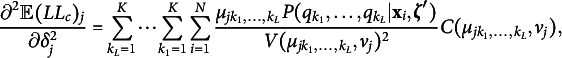

where (19)

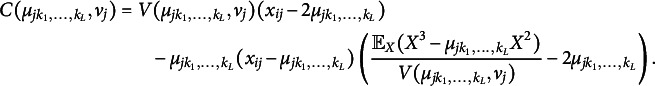



### 2.5 Latent trait covariance matrix

In the M2PCMPM EM algorithm (including the regularized variants), we assume the latent trait covariance matrix, 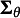 , fixed. The diagonal of 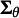 is fixed to the canonical value 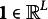 for identification purposes in this model with discrimination parameters—this is analogous to the identification assumption made in the unidimensional case in Beisemann (2022). A convenient choice for the off-diagonal is to assume orthogonal latent traits during estimation (i.e., fix all off-diagonal elements of 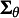 to 0). If the latent traits are in fact correlated, pronounced loadings of items on two or more factors can result. For the classical rotation approach, an oblique rotation can find a correlated solution with fewer items loading on two or more factors

In the case of strong(er) correlations between latent factors, this may put the regularized approach at a disadvantage as a sparse solution will not fit well when more than one loading is required to account for latent factor correlations. Sun et al. (2016) approach this problem by first estimating an unpenalized MIRT model to obtain latent factor correlation estimates from this model, which they plug into 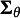 for the respective off-diagonal estimates. We use the same approach in this work, but we obtain the latent factor correlation from oblique rotation of the 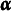 matrix. Note that an alternative would be to estimate the latent factor correlations within the EM algorithm, albeit this would require adjustments to the algorithm as well as the model identification constraints (compare Sun et al., 2016).

### 2.6 Computational aspects

The M2PCMPM EM algorithms are computationally expensive. Thus, we dedicated some effort to improving computational efficiency, as outlined below.

#### 2.7 Start values

In line with the start value approach Beisemann (2022) uses for the 2PCMPM, we set starting values for the M2PCMPM by fitting multidimensional two-parameter Poisson models to the data and compute starting values for the dispersion parameters as described in Beisemann (2022). Fitting these Poisson variants first saves computation time as each Poisson iteration of the EM algorithm is much less expensive than a CMP iteration, the obtained start values are already quite close to the CMP solution for the 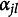 and the 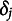 , and therewith reduce the number of required iterations of the M2PCMPM EM algorithm (compare Beisemann, 2022).

#### 2.8 Regularization tuning and warm starts

For the lasso-penalized M2PCMPM EM algorithm, the hyperparameter 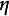 requires tuning to be optimally chosen. To this end, we use a grid of 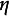 values to assess. Values of the grid are chosen equidistantly on the log scale (Hastie et al., 2009). To increase computational efficiency when fitting a penalized M2PCMPM for each 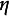 value on the grid, we implemented warm starts (Hastie et al., 2009), that is, we used the model parameter estimates of the previous model as start values for the subsequent model. To select the optimal 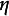 , one has to impose a criterion which 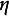 has to optimize. Traditionally, one may use cross-validation and optimize the RMSE of model predictions (Hastie et al., 2009). However, due to the high computational cost of the M2PCMPM EM algorithm and in line with prior research (Sun et al., 2016), we opted to use the Bayesian information criterion (BIC) as a criterion to optimize instead. Following Sun et al. (2016), for the lasso penalty, we computed the BIC (Schwarz, 1978) dependent on 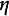 as (20)

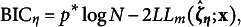

where 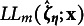 is the unpenalized marginal log-likelihood for the penalized model parameter estimates (using hyperparameter value 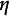 ), and 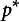 is the number of parameters 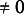 , i.e., the number of parameters for which the estimate is neither shrunken to 0 nor constrained to 0. We select the 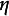 value minimizing 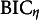 .

#### 2.9 Implementation

We implemented M2PCMPM EM algorithm (with and without penalities) in the R package countirt ( https://github.com/doebler/countirt ; please consult the package’s GitHub page for more information on the implementation and its limitations)^3^. For computational efficiency, the algorithm was implemented in R and C++, using among others the package GSL (Galassi et al., 2010), tied into R using Rcpp (Eddelbuettel et al., 2011). Multidimensional Gauss–Hermite quadrature was implemented using MultiGHQuad (Kroeze, 2016). For efficiency, quadrature grid truncation is used per default (i.e., quadrature points with very low quadrature weights are precluded from the grid).

## 3 Simulation study

In this small simulation study, we aimed to validate the proposed algorithms and illustrate the viability of their usage in different psychometric settings. The simulation study was run in R (R Core Team, 2023), using the package countirt to fit the M2PCMPMs. The code for the simulations and rds files of the saved simulation results are available at https://osf.io/n5792/?view_only=decbaf3ed16f4bac953ebc6be31c4859.

### 3.1 Design

In line with previous simulations regarding regularized item response models (Sun et al., 2016), we varied the number of latent traits between 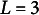 and 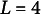 . Further, we varied the correlation between these latent traits ( 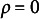 vs. 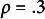 ). Choosing parameter values for a new class of count item response models is to a certain extent guess work and arbitrary. We tackled this issue by looking at what parameter value ranges occur at all for previously proposed and applied CMP-based count item response models (cf. Beisemann, 2022; Beisemann et al., 2024; Forthmann et al., 2020) and what sort of range we observed for the M2PCMPM on our example data. We did not use these exact values or any edge-case values from these studies and examples, but just used them to obtain a general feel for realistic parameter value ranges. The exact ranges and values were then chosen arbitrarily. For the model parameters, we used the same range of 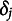 and 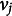 values across all conditions. For 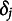 , we used values between 1.5 and 3.5, and for 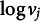 , we used values between 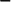 0.8 and 0.8 (i.e., implying—not very large—over- and underdispersion of varying degree), assigned randomly to the items. The true 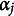 values depended on the simulation condition: Apart from the number of latent traits, we also varied the number number of items per trait ( 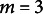 vs. 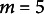 ). To the best of our knowledge, settings with small(er) numbers of items are realistic for count tests, with count tests often being comprised of less items than binary tests. We further varied the type of structure of the 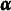 matrix (simple vs. slightly complex). With regard to the 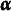 matrix structure, simple implies only single loadings of items on their assigned traits. The slightly complex structure implies in our simulation that a quarter of the items for each trait additionally—but to a lesser extent—load onto at least one of the other traits. We generated the 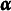 matrices as follows: (1) For each number-of-latent-traits and items-per-trait combination, we first set up a simple structure discrimination matrix, where each item loads onto only one trait. For each trait, there were *m* items associated with the respective traits. Per trait, for the respective *m* items, we chose the discriminations equidistantly between 0.2 and 0.3 and remaining discriminations were set to 0. (2) We then used the simple structure discrimination matrix set up in (1) to build the slightly complex structure. To this end, we kept any nonzero loadings as they were in (1). For any zero-loadings from (1), we sampled out of the values 
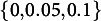
 with probabilities 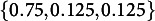 , so that we obtained some cross loadings. All true parameter values are displayed in the Supplementary Material. The described design factors were fully crossed to yield 16 simulation conditions (see Table 1). We ran 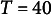 simulation trials per condition.^4^

**Table 1:** Overview of the 16 simulation conditions

*N*	*L*	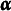 Structure	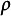	*m*
1200	3	Simple	0.00	3
1200	4	Simple	0.00	3
1200	3	Complex	0.00	3
1200	4	Complex	0.00	3
1200	3	Simple	0.30	3
1200	4	Simple	0.30	3
1200	3	Complex	0.30	3
1200	4	Complex	0.30	3
1200	3	Simple	0.00	5
1200	4	Simple	0.00	5
1200	3	Complex	0.00	5
1200	4	Complex	0.00	5
1200	3	Simple	0.30	5
1200	4	Simple	0.30	5
1200	3	Complex	0.30	5
1200	4	Complex	0.30	5

### 3.2 Data generation and model fitting

In each trial in each respective condition, we generated (inspired by our application example) 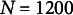 responses to 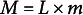 items under the M2PCMPM with the condition-specific model parameters. With regard to simulating item response data from the CMP distribution, we followed prior simulation studies on CMP-based item response models, using and adapting code from Forthmann et al. (2020) and Beisemann (2022). In each trial, we first fitted an exploratory M2PCMPM with upper-triangle identification constraint. The obtained solution was rotated once using the orthogonal varimax criterion(Kaiser, 1958, 1959) and once using the oblique oblimin (Clarkson & Jennrich, 1988), relying on the GPArotation package (Bernaards & Jennrich, 2005). Then, we fitted the lasso-penalized M2PCMPMs for hyperparameter tuning with regard to the BIC.^5^ We used a 12-value penalization grid of 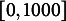 with values chosen equidistantly on the log scale (compare Hastie et al., 2009). We tuned the lasso-penalized M2PCMPMs once with the orthogonal latent trait assumption and once with a latent trait covariance matrix which incorporates the latent traits correlations obtained from the obliquely rotated M2PCMPM (see *Latent Trait Covariance Matrix*). All M2PCMPMs were fitted using the countirt package (see *Computational Aspects*).

We enhanced computational efficiency through several techniques. First, we used warm starts in tuning 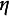 with regard to the BIC for the penalized M2PCMPMs (see *Computational Aspects*). Second, we used the parameter estimates obtained from the unpenalized exploratory M2PCMPM as start values for 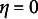 (which should result in immediate convergence as 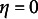 is the unpenalized case). Third, we adjusted the number of quadrature nodes per trait, in relation to the number of latent traits (with 10 nodes per trait for 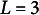 , and 4 nodes per trait with 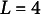 ).

### 3.3 Evaluation criteria

For the penalized M2PCMPMs, we evaluated the models for the 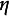 value selecting during hyperparameter tuning. Following Sun et al. (2016), we evaluated the correct estimation rate (CER), which we adapted to the upper-triangle identification constraint used here. The CER (adapted from Sun et al., 2016) is defined here as (21)

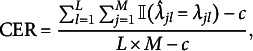

with *c* is the number of constraints imposed on 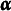 for identification, 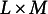 the number of elements in 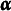 , and 
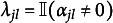
 and 
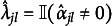
 , where 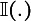 denotes the indicator function. Note that we defined the CER slightly differently than Sun et al. (2016) to better accommodate our identification constraint. The CER helps to assess whether the variable selection in the lasso-penalized models worked correctly, or to what extent. Performance of the BIC-based tuning for the lasso-penalized models was assessed by comparing the two 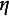 s selected by minimizing BIC and maximizing CER (Sun et al., 2016). We additionally report the number of elements in 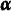 that were estimated to be zero (i.e., shrunken to 0) for the penalized M2PCMPMs.

Further, we assessed bias and RMSE for the intercept and (log-)dispersion parameters. As there are an infinite number of rotated solutions, bias and RMSE on each single discrimination parameter are less meaningful for rotated exploratory item response models. Based on the helpful suggestion of a reviewer, we evaluated the off-diagonal elements of 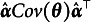 in terms of bias and RMSE (Battauz & Vidoni, 2022). This measure is identical for orthogonal and oblique rotations of loadings, so differences are only expected between the two lasso approaches and one of the rotations of the unpenalized models. The latter were indeed identical within numerical error.

### 3.4 Results

All trials were completed without any numerical instabilities and the EM algorithm(s) converged for all models in all trials and conditions. The average and median computation times across trials per condition are displayed in Table 2. Some of the conditions exceed 60h on the cluster available for this project, with some variance between randomly assigned computational nodes. We observed substantially lower times on an average laptop (up to several hours), so the computational times are provided for relative comparisons.

**Table 2:** Average (mean) and median (med) computation times (in seconds) for the different models in the 16 conditions

Condition	Rotate		Lasso (ortho)		Lasso (obli)	
	Mean	Med	Mean	Med	Mean	Med
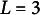 , Simple, 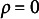 , 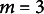	46283.61	45216.68	57253.41	54403.83	68838.00	68667.01
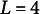 , Simple, 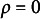 , 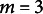	58324.05	53068.87	58462.86	50916.01	72311.45	56909.65
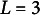 , Complex, 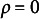 , 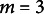	47302.36	47547.84	92965.29	85280.05	102951.27	99220.47
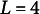 , Complex, 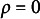 , 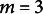	48059.90	36611.60	51141.60	49861.19	86535.05	76092.09
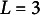 , Simple, 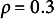 , 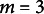	52099.54	51481.72	68512.43	67672.44	87602.04	83895.90
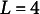 , Simple, 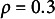 , 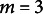	61413.70	42997.00	55539.63	56794.52	92688.53	84882.30
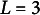 , Complex, 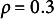 , 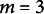	59194.82	60043.06	91861.01	87082.48	150744.07	144386.86
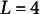 , Complex, 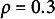 , 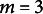	42349.63	33879.25	53489.87	51615.20	127026.13	115657.41
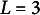 , Simple, 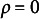 , 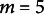	108298.80	97179.92	106282.62	100873.23	135246.50	130528.46
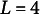 , Simple, 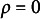 , 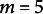	128602.85	103645.72	95848.96	89897.84	130119.02	114287.96
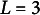 , Complex, 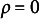 , 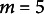	125037.58	103165.51	124297.37	106175.32	158021.63	142434.77
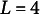 , Complex, 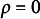 , 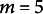	100351.37	85896.33	88783.71	77286.90	170419.37	161424.27
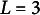 , Simple, 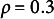 , 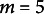	121959.14	102129.61	130078.07	124749.49	189087.04	175435.80
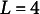 , Simple, 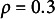 , 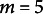	93551.76	77246.24	101061.76	90799.99	174620.27	155440.97
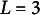 , Complex, 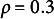 , 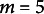	136516.21	128835.08	165670.78	167072.77	231389.87	206651.94
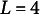 , Complex, 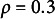 , 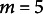	100963.79	88346.76	120748.39	121954.97	218278.40	208264.54

*Note*: Note that the lasso models include hyperparameter tuning, and thus multiple model fits in one instance, but for the first hyperparameter grid value, the rotate fit parameter estimates were used as start values (yielding a 1-iteration run of the algorithm). obli = oblique (latent traits are a priori assumed to be correlated). ortho = orthogonal (latent traits are a priori assumed to be orthogonal). *L* = number of latent traits. 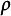 = true latent trait correlation. *m* = number of items per trait.

**Figure 1:**
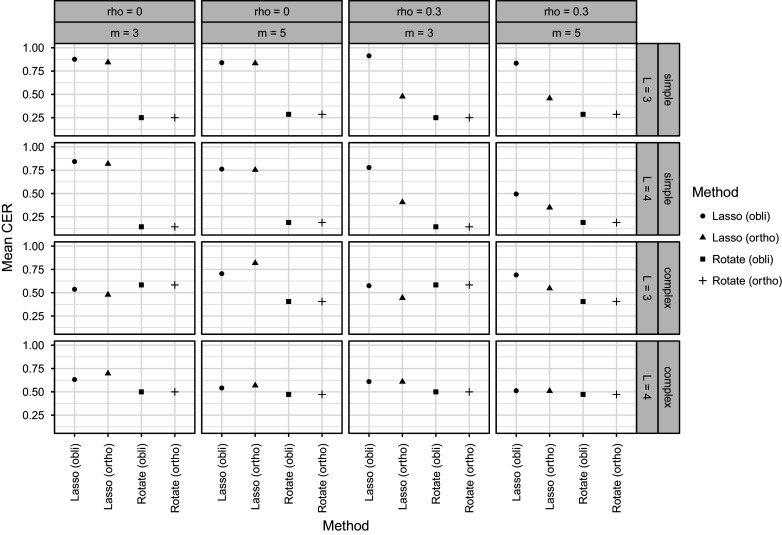
Mean Correct Estimation Rate (CER) estimates for each simulation condition. Estimates for the different model variants are shown on the *x*-axis and indicated by different shapes as detailed in the legend on the right-hand side. (L = number of latent tarits. r = true correlation between latent traits. m = number of items per trait. simple / complex = type of 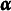 structure. Lasso / Rotate = model variant. ortho = orthogonal. obli = oblique.).

Figure 1 shows the average CER per condition and per method or model used. In the first two rows of Figure 1, we see the results for the simple 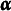 structure, and in the last two rows, the results for the complex 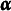 structure are displayed. There was a clear difference in performance between the two different 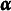 structures. For the simple 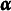 structure, in line with expectations, we see poor performance of the rotation methods (which are not able to shrink estimates down to exactly 0, putting them at a disadvantage in general in terms of CER). In conditions with complex 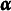 structure, the rotation methods performed better in these conditions as we would expect when there are fewer parameters that require shrinkage to exactly 0. In conditions with correlated latent traits, we can see that only the oblique lasso model showed decent performance (in most but not all conditions) in terms of CER. Especially for correlated latent traits, performance fell off for four latent traits in conjunction with five items per trait, even for the oblique lasso. For 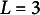 latent traits, more items per trait tended to increase performance (at least for complex 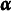 structure), but for 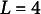 latent traits, more items tended to decrease performance (for both 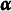 structures). One can again speculate that these last two observed patterns in the results might be due to the number of observations to number of parameters ratio which is considerably decreased for 4 traits and 5 items per trait.

**Figure 2:**
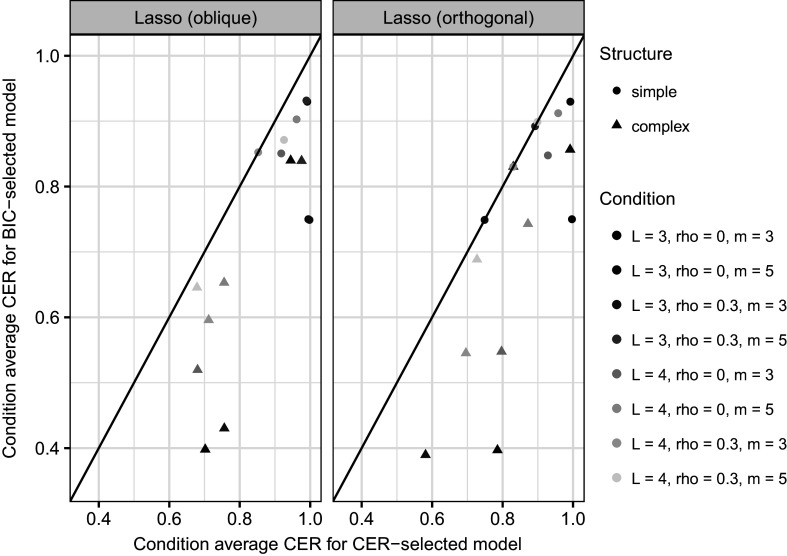
Condition average CER for the BIC-selected model (*y*-axis) against condition average CER for the CER-selected model (*x*-axis), shown in two separate panels (lasso with oblique latent covariance matrix on the left and lasso with orthogonal latent covariance matrix on the right). Simulation conditions (in terms of number of latent traits (L), latent factor correlation (r), and number of items per trait (m)) are shown in different colours as indicated by the legend on the right-hand side (under “Condition”). Different 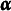 structures are represented by different shapes as indicated by the legend on the right-hand side (under “Structure”).

Figure 2 plots the (condition average) CER for the tuning parameter 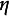 selected via the BIC (on the *y* axis) against the maximum (condition average) CER obtained by any of the models on the 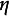 grid, i.e., the model we would have selected based on the CER. Figure 2 shows the two different lasso models in two separate panels. Figure 2 describes how well the BIC performed in terms of parameter tuning (Sun et al., 2016). Ideally, the BIC-selected 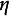 is the CER-selected 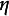 which would mean that the condition’s point in Figure 2 would lie on the diagonal black line. In Figure 2, we can see that this is the case for one condition for the oblique lasso ( 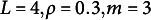 with simple 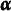 structure), and for four conditions for the orthogonal lasso ( 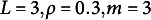 , 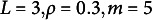 , 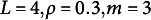 , and 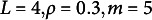 with simple 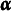 structure, and 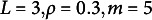 with complex 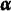 structure). For either method, conditions with simple 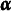 structure, more items, and/or more traits tended to exhibit better accuracy of BIC-based 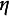 tuning with points in proximity of the line. For complex 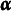 structure (compared to the other conditions), the CER were lower even when 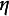 was selected based on the CER. Figure 2 shows here that for complex 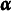 structure (compared to the other conditions), BIC-based tuning works notably better (with points closer to the diagonal line) for more items per trait (and even better if that is in conjunction with more latent traits).

Table 5 contains the average number and proportion of discrimination parameters shrunken to zero. Clearly, employing the 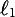 -penalty enforces a simple structure with 9% to 59% zeroes among the estimated discrimination parameters. In most situations, the oblique Lasso model is sparser, with more zeroes than the orthogonal model. The only exceptions to this are the cases with a complex structure of 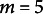 items per uncorrelated factor. These observations reinforce the conclusions based on the CER.

**Table 3:** Average bias (between-item 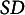 in parentheses) and RMSE (between-item 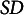 in parentheses) on 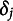 parameters across all items per condition

Design				Bias ( 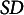 )			RMSE ( 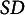 )		
*L*	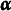 structure	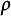	*m*	Lasso (obli)	Lasso (ortho)	Rotate	Lasso (obli)	Lasso (ortho)	Rotate
3	Simple	0	3	0.001 (0.002)	0.001 (0.002)	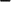 0.001 (0.002)	0.011 (0.002)	0.011 (0.002)	0.011 (0.002)
3	Simple	0	5	0.002 (0.003)	0.002 (0.003)	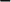 0.001 (0.002)	0.012 (0.004)	0.012 (0.004)	0.012 (0.004)
3	Simple	.3	3	0.002 (0.002)	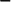 0.000 (0.001)	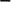 0.001 (0.001)	0.012 (0.003)	0.012 (0.003)	0.012 (0.003)
3	Simple	.3	5	0.003 (0.003)	0.000 (0.002)	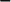 0.001 (0.002)	0.014 (0.004)	0.013 (0.004)	0.013 (0.004)
3	Complex	0	3	0.002 (0.002)	0.002 (0.002)	0.001 (0.002)	0.011 (0.002)	0.011 (0.002)	0.011 (0.002)
3	Complex	0	5	0.000 (0.002)	0.002 (0.002)	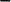 0.000 (0.002)	0.013 (0.004)	0.013 (0.004)	0.013 (0.004)
3	Complex	.3	3	0.003 (0.001)	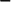 0.000 (0.001)	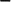 0.001 (0.001)	0.013 (0.003)	0.012 (0.003)	0.012 (0.003)
3	Complex	.3	5	0.002 (0.002)	0.000 (0.002)	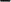 0.000 (0.002)	0.014 (0.003)	0.012 (0.003)	0.012 (0.003)
4	Simple	0	3	0.006 (0.005)	0.006 (0.004)	0.002 (0.003)	0.013 (0.004)	0.014 (0.003)	0.013 (0.002)
4	Simple	0	5	0.006 (0.002)	0.009 (0.003)	0.004 (0.002)	0.014 (0.003)	0.016 (0.003)	0.015 (0.003)
4	Simple	.3	3	0.008 (0.006)	0.005 (0.004)	0.003 (0.003)	0.015 (0.005)	0.014 (0.003)	0.013 (0.003)
4	Simple	.3	5	0.007 (0.003)	0.006 (0.002)	0.005 (0.002)	0.015 (0.003)	0.014 (0.002)	0.014 (0.002)
4	Complex	0	3	0.005 (0.003)	0.005 (0.004)	0.003 (0.003)	0.014 (0.004)	0.013 (0.004)	0.013 (0.004)
4	Complex	0	5	0.006 (0.002)	0.008 (0.002)	0.005 (0.002)	0.015 (0.003)	0.016 (0.003)	0.014 (0.002)
4	Complex	.3	3	0.007 (0.002)	0.005 (0.003)	0.005 (0.003)	0.015 (0.003)	0.014 (0.003)	0.014 (0.003)
4	Complex	.3	5	0.003 (0.003)	0.005 (0.003)	0.004 (0.003)	0.018 (0.003)	0.017 (0.004)	0.016 (0.004)

*Note*: Note that rotated models have the same 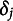 estimates regardless of rotation methods as those only affect 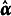 . obli = oblique (latent traits are a priori assumed to be correlated). ortho = orthogonal (latent traits are a priori assumed to be orthogonal). *L* = number of latent traits. 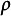 = true latent trait correlation. *m* = number of items per trait.

**Table 4:** Average bias ( 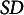 in parentheses) and RMSE ( 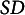 in parentheses) on 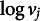 parameters across all items per condition

Design				Bias ( 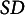 )			RMSE ( 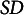 )		
*L*	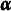 structure	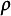	*m*	Lasso (obli)	Lasso (ortho)	Rotate	Lasso (obli)	Lasso (ortho)	Rotate
3	Simple	0	3	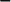 0.007 (0.013)	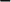 0.007 (0.014)	0.007 (0.017)	0.084 (0.029)	0.084 (0.029)	0.082 (0.030)
3	Simple	0	5	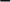 0.006 (0.009)	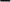 0.010 (0.027)	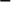 0.004 (0.031)	0.060 (0.018)	0.062 (0.022)	0.061 (0.022)
3	Simple	0.3	3	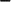 0.007 (0.014)	0.010 (0.012)	0.013 (0.013)	0.071 (0.020)	0.075 (0.025)	0.076 (0.026)
3	Simple	0.3	5	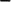 0.013 (0.022)	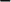 0.002 (0.020)	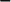 0.001 (0.022)	0.061 (0.023)	0.060 (0.019)	0.060 (0.019)
3	Complex	0	3	0.006 (0.013)	0.012 (0.015)	0.015 (0.015)	0.075 (0.023)	0.076 (0.022)	0.077 (0.022)
3	Complex	0	5	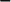 0.005 (0.008)	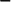 0.010 (0.021)	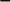 0.005 (0.019)	0.056 (0.013)	0.058 (0.017)	0.055 (0.014)
3	Complex	0.3	3	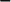 0.007 (0.012)	0.011 (0.010)	0.013 (0.011)	0.068 (0.018)	0.074 (0.025)	0.075 (0.024)
3	Complex	0.3	5	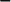 0.014 (0.019)	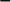 0.001 (0.012)	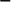 0.000 (0.011)	0.059 (0.018)	0.056 (0.015)	0.056 (0.014)
4	Simple	0	3	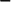 0.076 (0.148)	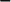 0.106 (0.214)	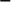 0.071 (0.165)	0.126 (0.134)	0.156 (0.194)	0.132 (0.144)
4	Simple	0	5	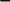 0.069 (0.095)	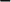 0.077 (0.104)	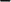 0.068 (0.106)	0.095 (0.087)	0.102 (0.096)	0.098 (0.095)
4	Simple	0.3	3	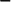 0.077 (0.142)	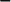 0.064 (0.147)	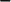 0.057 (0.138)	0.125 (0.124)	0.122 (0.129)	0.115 (0.120)
4	Simple	0.3	5	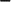 0.059 (0.098)	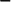 0.049 (0.088)	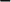 0.048 (0.089)	0.093 (0.088)	0.085 (0.075)	0.086 (0.075)
4	Complex	0	3	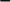 0.068 (0.135)	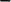 0.073 (0.209)	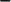 0.066 (0.206)	0.120 (0.122)	0.133 (0.186)	0.132 (0.182)
4	Complex	0	5	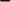 0.064 (0.093)	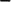 0.065 (0.093)	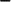 0.064 (0.096)	0.097 (0.081)	0.096 (0.081)	0.096 (0.082)
4	Complex	0.3	3	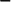 0.067 (0.146)	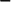 0.060 (0.173)	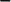 0.060 (0.173)	0.122 (0.131)	0.126 (0.151)	0.126 (0.150)
4	Complex	0.3	5	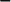 0.059 (0.080)	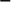 0.053 (0.076)	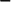 0.053 (0.076)	0.091 (0.071)	0.086 (0.064)	0.086 (0.064)

*Note*: Note that rotated models have the same 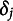 estimates regardless of rotation methods as those only affect 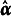 . obli = oblique (latent traits are a priori assumed to be correlated). ortho = orthogonal (latent traits are a priori assumed to be orthogonal). *L* = number of latent traits. 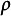 = true latent trait correlation. *m* = number of items per trait.

**Table 5:** Average number of discrimination parameters shrunken to zero by the 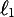 -penalty

Design				Lasso (orthogonal)			Lasso (oblique)		
*L*	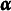 structure	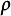	*M*	Mean	SD	Prop	Mean	SD	Prop
3	Simple	0.0	3	14.22	2.36	0.53	15.03	2.44	0.56
4	Simple	0.0	3	28.38	6.41	0.59	29.40	3.05	0.61
3	Complex	0.0	3	3.98	1.14	0.15	5.88	1.49	0.22
4	Complex	0.0	3	11.62	1.78	0.24	10.90	2.84	0.23
3	Simple	0.3	3	5.42	1.30	0.20	15.93	1.99	0.59
4	Simple	0.3	3	11.10	2.59	0.23	26.73	6.72	0.56
3	Complex	0.3	3	3.80	0.85	0.14	6.90	2.10	0.26
4	Complex	0.3	3	6.72	0.75	0.14	9.07	2.77	0.19
3	Simple	0.0	5	23.00	3.33	0.51	23.25	2.45	0.52
4	Simple	0.0	5	42.48	9.07	0.53	42.85	6.34	0.54
3	Complex	0.0	5	17.35	4.46	0.39	13.25	3.79	0.29
4	Complex	0.0	5	13.07	3.73	0.16	11.03	4.47	0.14
3	Simple	0.3	5	7.20	2.27	0.16	23.05	3.02	0.51
4	Simple	0.3	5	11.90	3.39	0.15	23.23	12.94	0.29
3	Complex	0.3	5	5.92	2.07	0.13	12.75	4.84	0.28
4	Complex	0.3	5	7.38	1.33	0.09	8.82	2.56	0.11

Bias and RMSE estimates for the rotation-invariant item parameters ( 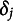 ’s and 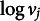 ’s) are shown in Tables 3 and 4, respectively. We can see that the intercept parameters can be estimated very well with very little bias (Table 3). For the dispersion parameters, we have (slightly) larger bias and RMSE estimates (Table 4), but overall still mostly satisfactory performance for a sample of 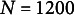 . In particular for 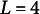 traits, performance is better for larger *m*, that is, for more items per trait. Settings with 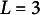 traits yielded better performance than those with 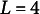 , likely as the number of observations to number of items ratio is smaller in the latter case for constant 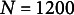 .

**Figure 3:**
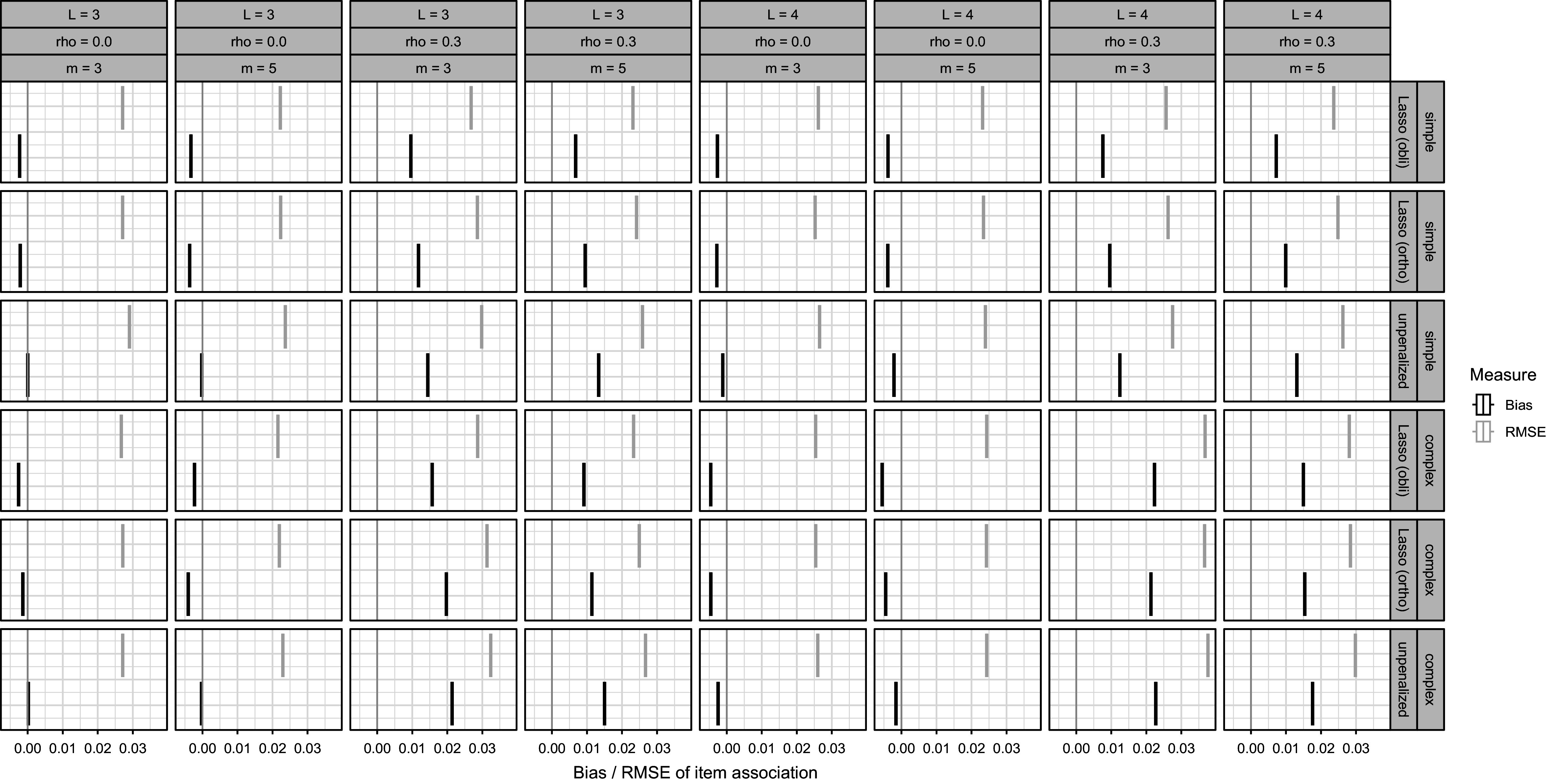
Bias and RMSE of the off-diagonal elements of 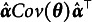 , a measure of association of item pairs.

Figure 3 investigates bias and RMSE in estimated item pair associations via the off-diagonal elements of 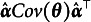 . Bias is negligible if 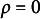 , but for 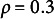 , slight biases become apparent, especially if the true discrimination parameter matrix is complex. As can be expected, the increase in Bias also increases the RMSE. In some scenarios (simple, 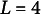 , 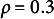 , 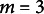 or 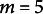 ), the oblique Lasso has somewhat lower Bias and RMSE.

## 4 Application example

To illustrate the application of an exploratory M2PCMPM together with a comparison of the two regularization based approaches with the traditional rotation based approach, we reanalyze data ( 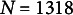 adolescents, including 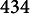 adolescents diagnosed as highly gifted) from a German intelligence test (*Berliner Intelligenzstrukturtest für Jugendliche: Begabungs- und Hochbegabungsdiagnostik*, BIS-HB; Jäger et al., 2006). The BIS-HB is an operationalization of the Berlin model of intelligence structure (Jäger, 1967, 1982, 1984). In line with this model, the BIS-HB assesses intelligence across four operational abilities (each measured in three content domains: figural, verbal, and numerical): processing capacity, creativity, memory, and processing speed. We reanalyze the responses for the two operational abilities, creativity and processing speed, which generate count responses. Processing speed is assessed using nine items (also reanalyzed in Doebler et al., 2014), creativity (in terms of idea flexibility) with five.

In our re-analysis, we investigate in how far we can recover the theoretical factor structure of two latent traits in an exploratory M2PCMPM. We fit the two variants (i.e., lasso and rotation) of the exploratory two-factor M2PCMPM with the upper-triangle identification constraint to the data and 12 quadrature nodes per trait, using the countirt package (see *Computational Aspects*). For the M2PCMPM in conjunction with rotation, we used an orthogonal varimax (Kaiser, 1958, 1959) and an oblique oblimin rotation (Clarkson & Jennrich, 1988). For the lasso-penalized M2PCMPM, we fitted one model with a priori orthogonal (i.e., uncorrelated) latent factors and one with a priori oblique (i.e., correlated) latent factors. For the latter, latent factor correlations obtained from the obliquely rotated M2PCMPM were used (compare Sun et al., 2016). We tuned the lasso-penalized M2PCMPMs using a 20-value penalization grid of 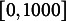 with values chosen equidistantly on the log scale (cf. Hastie et al., 2009) and used warm starts in 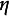 -tuning (see *Computational Aspects*). As in the simulation study, start values for the first M2PCMPMs on the tuning grid (i.e., for 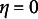 ) were the parameter estimates from the unpenalized M2PCMPM (before rotation).

The results are shown in Table 6. While we do not obtain a pattern of perfect 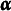 simple structure for any of the methods, we can see that in particular for the approaches with oblique latent traits, the estimates for the 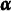 matrix align well with theoretical considerations. That is, for the oblimin-rotated unpenalized M2PCMPM, we can see that the processing speed items load mostly on the first trait (i.e., processing speed), while the creative thinking items load mostly on the second trait (i.e., creative thinking). Only the processing speed items BD and OE load overall rather weakly onto either factor, with a small preference for the processing speed factor. A similar pattern of results emerged for the lasso-penalized M2PCMPM with oblique latent traits, with the penalty-imposed shrinkage amplifying the theoretically implied loading structure further. For the creative thinking items AM and ZF as well as for the processing speed item UW, the discrimination parameters were even shrunken to 0. We can see that the assumption that the latent traits are uncorrelated (i.e., varimax-rotated unpenalized M2PCMPM and lasso-penalized M2PCMPM with orthogonal latent traits) yielded a less differentiated loading structure, in particular for the creative thinking items which still load highest onto the second trait but also less negligibly onto the first, especially for the lasso-penalized M2PCMPM with orthogonal latent traits. Intercept ( 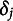 ) and log-dispersion ( 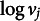 ) estimates were—as we would expect—very similar across methods. Note the rotated M2PCMPMs have only one set each as they are both based on the same unpenalized M2PCMPM for which we only rotate the 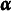 matrix, leaving the other parameters unchanged. Items exhibited a mix of over- and underdispersion, with some even close to equidispersion (i.e., 0 for 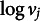 as 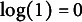 ), highlighting the strength of the CMP distribution to account for such a variation of dispersion across items.

**Table 6:** Results example (Processing speed (P) and creativity (C))

Item		RZ (P)	IT (C)	SI (P)	XG (P)	UW (P)	TG (P)	KW (P)	ZS (P)	BD (P)	OE (P)	AM (C)	ZF (C)	EF (C)	OJ (C)
Method	Parameter														
varimax	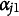	0.262	0.114	0.219	0.258	0.325	0.219	0.157	0.139	0.080	0.091	0.095	0.079	0.104	0.097
	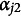	0.067	0.192	0.099	0.042	0.117	0.064	0.062	0.055	0.049	0.053	0.272	0.212	0.253	0.189
oblimin	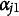	0.278	0.046	0.212	0.284	0.329	0.228	0.156	0.138	0.071	0.082	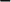 0.013	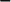 0.004	0.006	0.027
	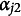	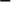 0.013	0.193	0.042	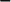 0.041	0.026	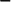 0.000	0.020	0.018	0.031	0.032	0.296	0.228	0.269	0.195
Lasso (ortho)	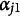	0.261	0.154	0.229	0.250	0.335	0.222	0.163	0.145	0.087	0.099	0.155	0.126	0.158	0.138
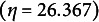	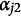	0	0.150	0.033	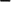 0.026	0.019	0.000	0.014	0.012	0.023	0.022	0.231	0.175	0.212	0.149
Lasso (obli)	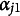	0.256	0.050	0.208	0.270	0.333	0.225	0.161	0.140	0.072	0.086	0.000	0.000	0.007	0.026
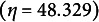	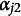	0	0.172	0.029	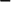 0.040	0.000	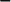 0.009	0.004	0.005	0.024	0.020	0.266	0.213	0.251	0.183
varimax /	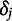	2.369	1.780	3.580	2.747	3.069	2.405	3.223	3.460	3.944	3.486	1.313	1.547	1.576	1.491
oblimin	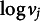	0.282	0.815	-1.131	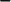 0.095	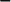 0.436	0.566	0.510	0.009	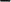 0.117	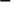 0.021	0.787	0.909	0.627	0.993
Lasso (ortho)	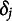	2.370	1.781	3.580	2.748	3.069	2.405	3.223	3.460	3.944	3.486	1.314	1.548	1.577	1.492
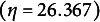	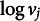	0.271	0.814	-1.134	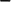 0.108	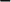 0.434	0.564	0.515	0.014	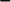 0.119	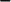 0.021	0.793	0.898	0.636	0.988
Lasso (obli)	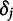	2.358	1.773	3.570	2.737	3.053	2.394	3.215	3.453	3.939	3.481	1.307	1.540	1.569	1.484
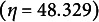	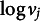	0.264	0.799	-1.141	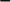 0.117	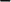 0.416	0.573	0.540	0.015	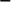 0.120	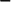 0.022	0.770	0.911	0.623	0.998

*Note*: Factor correlation from oblique rotation (oblimin): 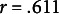 . Identification constraints are printed in gray. obli = oblique (a priori correlated latent factors). ortho = orthogonal (a priori uncorrelated latent factors).

## 5 Discussion

This work proposes a novel multidimensional count item response model with flexible dispersion modeling: the multidimensional two-parameter Conway–Maxwell–Poisson model (M2PCMPM). A number of existing count item response models (Beisemann, 2022; Forthmann et al., 2018, 2020; Myszkowski & Storme, 2021; Rasch, 1960) can be understood as special cases of the M2PCMPM, rendering the M2PCMPM a general overarching model class. The M2PCMPM can be employed in an exploratory manner—which this work primarily focused on—but also in a confirmatory manner by imposing constraints on model parameters. As a consequence, even more special cases of count item response models can be obtained and formulated as well as estimated within the M2PCMPM framework. We derived marginal maximum likelihood estimation methods based on the EM algorithm (Dempster et al., 1977). For exploratory M2PCMPMs, we investigated using rotation methods (e.g., Carroll, 1957; Clarkson & Jennrich, 1988; Kaiser, 1958, 1959) in conjunction with the proposed M2PCMPM-EM algorithm for obtaining a simple structure solution for the discrimination parameter matrix. Alternatively, we developed a 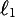 -penalized (i.e., lasso-penalized; Tibshirani, 1996) variant of the M2PCMPM-EM algorithm, which can be used to the same end. We explored versions of this algorithm with a priori uncorrelated latent traits and with a priori correlated latent traits. In a simulation study and an application example, we assessed and compared the two proposed algorithms for fitting exploratory M2PCMPMs.

### 5.1 Performance patterns from the simulation study

The conducted simulation study showed stable numerical performance for the developed algorithms in the investigated simulation settings. Bias and RMSE on the intercept and (log) dispersion parameters were overall satisfactory, with differences in performance between conditions in line with prior research on CMP-based count item response models (Beisemann, 2022; Beisemann et al., 2024). In conditions with more latent traits, we tended to observe more bias, in particular for the (log) dispersion parameters. Due to rotational indeterminacy, we assessed bias and RMSE of discrimination parameter estimates with a measure of item level associations. For a number of the conditions, we observed decent performance, with the oblique lasso approaches slightly better. Conditions in which bias and RMSE were somewhat pronounced were those with correlated traits.

In terms of BIC-based hyperparameter tuning for the lasso-penalized M2PCMPM-EM algorithm (with either a priori correlated or a priori uncorrelated latent factors), we found performance differed notably depending on the condition. Assessing tuning performance following Sun et al. (2016), we found that performance was in general better for an underlying simple structure of the 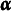 matrix. Unsurprisingly, more complex structures of the 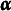 matrix were more challenging as these are less clearly variable selection problems. With more items and/or more traits, the accuracy of the BIC-based hyperparameter tuning tended to improve. Compared to Sun et al. (2016)’s assessment of BIC-based hyperparameter tuning for lasso-penalized binary models, we observed overall (more or less pronounced) worse performance for count models (not just of the BIC tuning, but also of the CER-based tuning which is perhaps surprising at first glance). It is worth pointing out that the direct comparison to the models in Sun et al. (2016) is not entirely appropriate as Sun et al. (2016) defined the CER slightly differently to us (see above). The observed pattern may also be confounded with the number of penalized parameters—in our simulation, the smallest setting only included nine items, which leaves (with identification constraints) only six freely estimated, penalized parameters. In this instance, one misclassification already equates to a change of 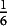 in the CER. As discussed further below, these results suggest that while the BIC-based hyperparameter tuning appears to work decently for some conditions, hyperparameter tuning for the lasso-penalized M2PCMPM-EM algorithm could still be improved by future research. These results also suggest that future research might wish to consider alternatives to the CER for performance evaluation. For example, one could extract the model-implied item covariance matrix and compare it to the observed item covariance matrix using matrix norms.

### 5.2 Limitations and further avenues for future research

Our simulation study was designed to provide a proof of concept for the proposed model and algorithms. It is important to note that it should only be regarded as such, and that it has severe limitations with regard to the number of simulation trials, the number of quadrature nodes per traits, and the sample size investigated. Please be aware that small numbers of simulation trials and low numbers of quadrature nodes per trait run the risk of yielding inaccurate or at least imprecise results. As a proof-of-concept type simulation, and as guided by previous research (Sun et al., 2016), it focused on scenarios with three or four latent traits. Future research could explore higher dimensional scenarios. In such settings, the Gauss-Hermite quadrature based M2PCMPM EM algorithm is likely going to reach its limitation, as Gauss-Hermite quadrature is known not to scale well to high-dimensional problems (Chalmers, 2012). Thus, future research in this regard could explore alternative integral approximations, such as Monte Carlo based methods. Further, the maximum test length investigated in our simulation study was 20 items. Future research could investigate more extensive tests, including more items. This should assess the models’ viability for use with larger tests.

In light of the limitations of the presented simulation study and upon a reviewer’s helpful suggestion, we attempted to conduct a more extensive simulation study, with 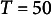 simulation trials per condition, setting the quadrature nodes per trait for conditions with 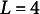 traits up to 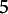 , and including eight additional conditions with a sample size of 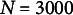 . A full overview of the design is available in the Supplementary Material. We ran into a computational issue: A number of conditions ran out of wall time (maximal wall time on the cluster used was 28 days) before they could be completed. This concerned primarily but not exclusively the conditions with 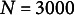 . The Supplementary Material contains an overview over which conditions were completed. Results are very similar and suggest that the number of quadrature nodes was not too low to obtain meaningful results reported here. The additional results (for those conditions for which all 50 trials were completed within the maximum wall time) are depicted in the Supplementary Material. We encourage future research to attempt a more comprehensive simulation, including more simulation trials and higher numbers of quadrature nodes per trait, especially if they have access to more computation power. Any definitive recommendations on using the proposed models should be held off until such a comprehensive investigation has been conducted.

We implemented the proposed algorithms in R and C++ within the countirt package. To this end, we built upon implementations of the 2PCMPM (Beisemann, 2022) and related models (Beisemann et al., 2024) in countirt. These implementations all use a naive interpolation-from-grid approach for some of the CMP distribution-related quantities to stabilize, facilitate and fasten computations. This approach worked well in our simulation study and its settings, but can be expected to work less well in settings where the data do not align well with the interpolation grid (see https://github.com/doebler/countirt for details). In a regression framework, Philipson and Huang (2023) developed a sophisticated and theory-based interpolation approach for CMP models, which allows not only inter- but also extrapolation from a specifically designed grid. Future research could aim to apply and extend their work to the (multidimensional) IRT context for CMP models.

In any case, our implementation in countirt can certainly be improved upon in terms of computational efficiency. We encourage future research to develop faster and more computationally efficient implementations of our proposed algorithms. We would expect that only such advances would allow the proposed models to be used in big data settings, such as big-data processing data applications. Future research could investigate what advances could make this possible to what extent.

For comparability with the rotation approach and for computational reasons, we did not tune our lasso penalty term on a training data set. However, for regularization methods that would be the recommended approach (Hastie et al., 2009) and is what we would recommend for high-stakes applications. This approach should prohibit overfitting to the data more aptly. In general, our tuning for the lasso penalty term simply used a grid with equidistant tuning parameter values on the log-value space (as is typically recommended; Hastie et al., 2009) and was based on the BIC. As we saw in the simulation study results, for certain settings, the selection of the tuning parameter could still be improved. In fact, sometimes the correct estimation rates were even low when they were used to choose the tuning parameter value. Future research might research how parameter tuning can be improved for the M2PCMPM lasso-EM algorithm and what computationally equally economical alternatives to the BIC as a tuning criterion could be used. Further, more investigation of tuning and the tuning grid used could also be interesting and helpful. Such investigations are going to have to face the computation time challenge that these computationally expensive models pose. Other than the warm starts already used in this work, other avenues such as EM algorithm accelerators might be explored (see Beisemann et al., 2020, for a recent overview of state-of-the-art methods).

For the penalization, we focused on the lasso (Tibshirani, 1996) which aligns with other research on penalization in item response models (Cho et al., 2022; Sun et al., 2016). Future research could explore the relaxed lasso (Meinshausen, 2007) to avoid shrunken nonzero estimates. Lasso penalization is also known to perform less well in settings with correlated variables (Hastie et al., 2009), which corresponds to latent factor correlations in item response model settings. Future research could address such limitation by extending the lasso-penalized M2PCMPM EM algorithm to penalties such as the elastic net (Zou & Hastie, 2005), which adaptively combines properties of the lasso and the ridge (Hoerl & Kennard, 1970) penalty. Alternative penalties such as the smoothly clipped absolute deviation (SCAD; Fan & Li, 2001) could also be explored (for an application of SCAD in IRT, see e.g., Robitzsch, 2023). Other ways in which the penalized algorithms themselves could be extended by future research would be, for example, the incorporation of latent factor correlation estimation into the algorithm, rather than the two-step method by Sun et al. (2016) that we used here to have the algorithm account for correlated factors.

Finally, the M2PCMPM framework proposed in this work can also in itself be a stepping stone for future research. That is, the M2PCMPM framework offers researchers the opportunity to propose, fit, and investigate a number of new count item response models that can be accomodated by the M2PCMPM framework as special cases. This can be achieved by exploring the confirmatory side of the M2PCMPM framework which the present work only briefly touched on. Future research could suggest new constraints through which new count item response models can be obtained from the M2PCMPM. Furthermore, for the M2PCMPM framework to be complete and applicable in practice, it needs to be enriched in the future by developing multi-group and differential item functioning extensions within the framework as well as by deriving person parameter estimators, item fit, and person fit measures. We propose the following tentative recommendations for utilizing the M2PCMPM: (1) Unlike binary and ordinal IRT models, fewer items per factor are required, as item information can be substantially higher in the count data case (Beisemann et al., 2020).In fact, as few as three items per factor may suffice, particularly if a simple factor structure is plausible and the discrimination parameters are sufficiently large. Based on our experience with several authentic datasets, we suggest starting with a linear factor analysis of log-counts. (2) When computational resources permit, oblique or orthogonal Lasso methods should be considered, as the resulting zeroes in the discrimination parameter matrix facilitate interpretation. The BIC criterion is our recommended method for tuning the Lasso. (3) A limitation of the Lasso approach, however, is that the resulting models cannot be freely rotated. (4) If excess zeroes are observed in the count data (zero-inflation) the CMP distribution is not adequate. Similarly, once the counts are very large or the over- or underdispersion becomes too extreme, numerical problems can arise. More research on extensions to other distributions are needed for these cases.

## Supporting information

Beisemann et al. supplementary material Beisemann et al. supplementary material

## Data Availability

The algorithms developed in this work have been implemented in the R package countirt (https://github.com/mbsmn/countirt/tree/multidimensional). The R code for the simulation study and the R data files of the simulation results are available on OSF (https://osf.io/n5792/). The data set re-analyzed for the example could not be made publicly available as this was guaranteed to participants at the time of data collection during the original study.

## Footnotes

1 For consistency and readability, we use a parameterization and notation here which is going to most easily generalize to the multidimensional case in the following sections. The original parameterization by Rasch (1960) is not log-linear but multiplicative.

2 Following our understanding of the notation in Sun et al. (2016), in each iteration of (1)(b)(i) in Algorithm 1 in Appendix B, we update  one-step late in (ii’). That is, we update  in (i’) on the basis of the at that point most up-to-date  , but use the previous  in (ii’). Please compare the appendix in Sun et al. (2016).

3 At the time of writing this manuscript, the M2PCMPM-related algorithms are implemented on multidimensional branch: https://github.com/doebler/countirt/tree/multidimensional . In the future, this branch is going to be merged into the main branch.

4 Note that with these models and the hyperparameter tuning for the regularization, each trial is computationally very expensive. For computational feasibility and as we simulated for a large sample of  , we were only able to run  simulation trials. This is in line with prior research (e.g., Sun et al., 2016 ran only 50 trials).

5 Here, we opted for fitting the penalized models for the different  values on the entire data set and selected the best fitting one. This approach is more comparable to the rotated models. However, note that in the machine learning literature, it would be preferred to tune the hyperparameter first on a training data set (i.e., a subsample of the sample) and then fit the model with the selected  on the remaining test data set. The latter approach will be less prone to overfitting than the first.

## Supplementary material

The supplementary material for this article can be found at https://doi.org/10.1017/psy.2024.17.

## APPENDICES

### A First derivative of the CMP variance

For the second derivatives in terms of  and  in Equations 17–18, we need the derivative of the variance *V* in terms of  and  . That is,

(A.1)

(A.2)

and

(A.3)

(A.4)

The first equality in both equation holds because for any random variable *W* it holds that  . Taking the derivative of  with regard to  and  is trivial. To take the derivative of  with regard to  and  , we used results provided in Huang (2017) and derivation rules.

## C

### C.1 Special cases

The M2PCMPM contains a number of count data item response models as special cases. For  , the M2PCMPM simplifies to the 2PCMPM (Beisemann, 2022) and with the additional constraint that  the model further simplifies to the Conway–Maxwell–Poisson Counts Model (CMPCM; Forthmann et al., 2020). For  , but equal slope parameters across items and traits, the M2PCMPM simplifies to a multidimensional CMPCM. Whenever all item-specific dispersions are fixed to be equal to 1 (i.e.,  ), the CMP density simplifies to the Poisson density. Consequently, the M2PCMPM also contains the RPCM (Rasch, 1960), the Two-Parameter Poisson Counts Model (2PPCM; Myszkowski & Storme, 2021), and multidimensional extensions of the RPCM and the 2PPCM (Forthmann et al., 2018; Myszkowski & Storme, 2021). Thereby, the M2CMPM offers the possibility of a comprehensive framework for count data item response modeling which subsumes a number of existing count data item response models.

### C.2 Confirmatory models by imposing constraints

While not a focus of the present work, we wanted to note that with the M2PCMPM EM algorithm, one can also fit confirmatory multidimensional count data item response models. That is, one can impose constraints on the item parameters (in particular but not exclusively, the slope parameters) and evaluate the specified model’s fit to the data. Confirmatory models should be identified by the imposed constraints. For instance, the fit of a perfect simple structure to the data can be evaluated by imposing constraints which imply single loadings of each item onto only one trait *l* (for a fixed *l*) of the latent traits, respectively, and   .
