# Supplementary material for: Every Trait Counts: Marginal Maximum Likelihood Estimation for Novel Multidimensional Count Data Item Response Models with Rotation or [image] –Regularization for Simple Structure
Source: Psychometrika. 2025 Jan 3;90(1):304–30. doi: 10.1017/psy.2024.17 (PMC12478621; doi:10.1017/psy.2024.17)
Supplement: Beisemann et al. supplementary material [file S0033312324000176sup001.pdf]

Online Supplement: Every Trait Counts: Marginal Maximum Likelihood Estimation for Novel Multidimensional Count Data Item Response Models with Rotation or ℓ_1–Regularization for Simple Structure

Blinded for Review

1 True Parameter Values for the Simulation Study

For the δ_j parameters, we used the following true values (in the order of item numbers, i.e., the first value corresponds to the true parameter of item 1, etc.). Values were rounded to second decimal point here for display. Exact values can be generated as detailed in the `R` script on OSF (`https://osf.io/n5792/`).

1. For 9 items: {2.00, 3.25, 1.75, 2.25, 3.00, 1.50, 3.50, 2.50, 2.75}
2. For 12 items: {1.68, 2.41, 3.32, 2.59, 3.14, 3.50, 1.50, 2.77, 2.05, 2.95, 1.86, 2.23}
3. For 15 items: {2.64, 3.36, 3.21, 1.93, 2.79, 2.36, 1.64, 3.07, 1.79, 2.50, 1.50, 2.21, 2.07, 3.50, 2.93}
4. For 20 items: {2.55, 2.03, 2.34, 1.92, 2.97, 1.71, 1.50, 2.87, 3.18, 2.45, 3.50, 3.08, 3.29, 2.66, 2.24, 2.76, 1.61, 3.39, 2.13, 1.82}

For the ν_j parameters, we used the following true values (in the order of item numbers, i.e., the first value corresponds to the true parameter of item 1, etc.). Values were rounded to second decimal point here for display. Exact values can be generated as detailed in the `R` script on OSF (`https://osf.io/n5792/`).

1. For 9 items: {0.82, 0.55, 2.23, 0.67, 1.00, 1.82, 1.22, 0.45, 1.49}
2. For 12 items: {1.08, 1.66, 2.23, 1.92, 1.44, 0.80, 0.93, 0.70, 1.24, 0.52, 0.45, 0.60}
3. For 15 items: {1.00, 0.56, 0.71, 1.41, 0.63, 1.12, 0.45, 1.99, 0.50, 0.89, 0.80, 2.23, 1.77, 1.58, 1.26}
4. For 20 items: {1.88, 0.96, 0.45, 1.59, 0.88, 0.68, 0.74, 2.23, 0.53, 0.49, 0.63, 1.46, 1.34, 0.81, 1.23, 1.04, 0.58, 2.05, 1.13, 1.73}

For the α_{jl} parameters, we used the following true values (for each trait in the order of item numbers, i.e., the first value corresponds to the true parameter of item 1, etc.).

1. For 9 items:
   (a) Simple structure: Trait 1: {0.20, 0.25, 0.30, 0.00, 0.00, 0.00, 0.00, 0.00, 0.00}, Trait 2: {0.00, 0.00, 0.00, 0.20, 0.25, 0.30, 0.00, 0.00, 0.00}, Trait 3: {0.00, 0.00, 0.00, 0.00, 0.00, 0.00, 0.20, 0.25, 0.30}
   (b) Slightly complex structure: Trait 1: {0.20, 0.25, 0.30, 0.00, 0.00, 0.10, 0.00, 0.00, 0.00}, Trait 2: {0.05, 0.00, 0.00, 0.20, 0.25, 0.30, 0.10, 0.00, 0.10}, Trait 3: {0.05, 0.05, 0.10, 0.10, 0.00, 0.00, 0.20, 0.25, 0.30}
2. For 12 items:

(a) Simple structure: Trait 1: {0.20, 0.25, 0.30, 0.00, 0.00, 0.00, 0.00, 0.00, 0.00, 0.00, 0.00, 0.00}, Trait 2: {0.00, 0.00, 0.00, 0.20, 0.25, 0.30, 0.00, 0.00, 0.00, 0.00, 0.00, 0.00}, Trait 3: {0.00, 0.00, 0.00, 0.00, 0.00, 0.00, 0.20, 0.25, 0.30, 0.00, 0.00, 0.00}, Trait 4: {0.00, 0.00, 0.00, 0.00, 0.00, 0.00, 0.00, 0.00, 0.00, 0.20, 0.25, 0.30}

(b) Slightly complex structure: Trait 1: {0.20, 0.25, 0.30, 0.00, 0.10, 0.10, 0.00, 0.05, 0.05, 0.10, 0.00, 0.00}, Trait 2: {0.10, 0.05, 0.00, 0.20, 0.25, 0.30, 0.00, 0.10, 0.00, 0.05, 0.05, 0.10}, Trait 3: {0.00, 0.00, 0.05, 0.00, 0.00, 0.10, 0.20, 0.25, 0.30, 0.00, 0.00, 0.00}, Trait 4: {0.00, 0.10, 0.00, 0.00, 0.05, 0.00, 0.00, 0.00, 0.00, 0.20, 0.25, 0.30}

3. For 15 items:

(a) Simple structure: Trait 1: {0.20, 0.22, 0.25, 0.28, 0.30, 0.00, 0.00, 0.00, 0.00, 0.00, 0.00, 0.00, 0.00, 0.00, 0.00}, Trait 2: {0.00, 0.00, 0.00, 0.00, 0.00, 0.20, 0.22, 0.25, 0.28, 0.30, 0.00, 0.00, 0.00, 0.00, 0.00}, Trait 3: {0.00, 0.00, 0.00, 0.00, 0.00, 0.00, 0.00, 0.00, 0.00, 0.00, 0.20, 0.22, 0.25, 0.28, 0.30}

(b) Slightly complex structure: Trait 1: {0.20, 0.22, 0.25, 0.28, 0.30, 0.00, 0.00, 0.00, 0.00, 0.00, 0.00, 0.00, 0.00, 0.00, 0.00}, Trait 2: {0.00, 0.05, 0.00, 0.10, 0.00, 0.20, 0.22, 0.25, 0.28, 0.30, 0.00, 0.00, 0.05, 0.05, 0.00}, Trait 3: {0.00, 0.00, 0.00, 0.00, 0.00, 0.00, 0.00, 0.05, 0.00, 0.00, 0.20, 0.22, 0.25, 0.28, 0.30}

4. For 20 items:

(a) Simple structure: Trait 1: {0.20, 0.22, 0.25, 0.28, 0.30, 0.00, 0.00, 0.00, 0.00, 0.00, 0.00, 0.00, 0.00, 0.00, 0.00, 0.00, 0.00, 0.00, 0.00, 0.00}, Trait 2: {0.00, 0.00, 0.00, 0.00, 0.00, 0.20, 0.22, 0.25, 0.28, 0.3, 0.00, 0.00, 0.00, 0.00, 0.00, 0.00, 0.00, 0.00, 0.00, 0.00}, Trait 3: {0.00, 0.00, 0.00, 0.00, 0.00, 0.00, 0.00, 0.00, 0.00, 0.00, 0.20, 0.22, 0.25, 0.28, 0.30, 0.00, 0.00, 0.00, 0.00, 0.00}, Trait 4: {0.00, 0.00, 0.00, 0.00, 0.00, 0.00, 0.00, 0.00, 0.00, 0.00, 0.00, 0.00, 0.00, 0.00, 0.00, 0.20, 0.22, 0.25, 0.28, 0.30}

(b) Slightly complex structure: Trait 1: {0.20, 0.22, 0.25, 0.28, 0.30, 0.00, 0.00, 0.00, 0.00, 0.00, 0.00, 0.10, 0.00, 0.05, 0.00, 0.00, 0.00, 0.00, 0.00, 0.00}, Trait 2: {0.00, 0.00, 0.00, 0.00, 0.05, 0.20, 0.22, 0.25, 0.28, 0.30, 0.05, 0.05, 0.00, 0.00, 0.00, 0.00, 0.00, 0.10, 0.00, 0.10}, Trait 3: {0.00, 0.00, 0.05, 0.00, 0.05, 0.00, 0.00, 0.05, 0.05, 0.00, 0.20, 0.22, 0.25, 0.28, 0.30, 0.00, 0.10, 0.00, 0.00, 0.00}, Trait 4: {0.00, 0.00, 0.05, 0.05, 0.10, 0.05, 0.10, 0.00, 0.10, 0.00, 0.10, 0.00, 0.10, 0.00, 0.05, 0.20, 0.22, 0.25, 0.28, 0.30}

2 Extended Simulation Study

2.1 Design

We extended the simulation study from the paper through the inclusion of eight additional simulation conditions with $N = 3000$. We obtained the following simulation design:

Condition	N	L	$\boldsymbol{\alpha}$ structure	Cor. latent traits	m
1	1200	3	simple	0.00	3
2	1200	4	simple	0.00	3
3	1200	3	complex	0.00	3
4	1200	4	complex	0.00	3
5	1200	3	simple	0.30	3
6	1200	4	simple	0.30	3
7	1200	3	complex	0.30	3
8	1200	4	complex	0.30	3
9	1200	3	simple	0.00	5
10	1200	4	simple	0.00	5
11	1200	3	complex	0.00	5
12	1200	4	complex	0.00	5
13	1200	3	simple	0.30	5
14	1200	4	simple	0.30	5
15	1200	3	complex	0.30	5
16	1200	4	complex	0.30	5
17	3000	3	simple	0.00	5
18	3000	4	simple	0.00	5
19	3000	3	complex	0.00	5
20	3000	4	complex	0.00	5
21	3000	3	simple	0.30	5
22	3000	4	simple	0.30	5
23	3000	3	complex	0.30	5
24	3000	4	complex	0.30	5

The extended simulation study was run with $T = 50$ simulation trials and 5 quadrature nodes per trait for conditions with $L = 4$ traits. Otherwise, the extended simulation study was run just like the smaller simulation study in the paper, where all other details are described.

We splitted each condition into two jobs on a computing cluster, each running 25 simulation trials in parallel using the `doParallel` package (Microsoft Corporation & Weston, 2022). Thus, each condition represents two jobs which were run on the computing cluster with a max wall time of 28 days. The following table gives an overview over which jobs were completed within the maximum wall time (compl. = completed):

Condition	N	L	$\boldsymbol{\alpha}$ structure	Cor. latent traits	m	Job 1 compl.	Job 2 compl.	Compl. trials
1	1200	3	simple	0.00	3	YES	YES	50
2	1200	4	simple	0.00	3	YES	YES	50
3	1200	3	complex	0.00	3	YES	YES	50
4	1200	4	complex	0.00	3	YES	YES	50
5	1200	3	simple	0.30	3	YES	YES	50
6	1200	4	simple	0.30	3	YES	YES	50
7	1200	3	complex	0.30	3	YES	YES	50
8	1200	4	complex	0.30	3	YES	YES	50
9	1200	3	simple	0.00	5	YES	NO	25
10	1200	4	simple	0.00	5	NO	YES	25
11	1200	3	complex	0.00	5	YES	YES	50
12	1200	4	complex	0.00	5	YES	YES	50
13	1200	3	simple	0.30	5	YES	YES	50
14	1200	4	simple	0.30	5	YES	YES	50
15	1200	3	complex	0.30	5	YES	YES	50
16	1200	4	complex	0.30	5	YES	YES	50
17	3000	3	simple	0.00	5	YES	YES	50
18	3000	4	simple	0.00	5	NO	NO	0
19	3000	3	complex	0.00	5	YES	NO	25
20	3000	4	complex	0.00	5	NO	NO	0
21	3000	3	simple	0.30	5	YES	YES	50
22	3000	4	simple	0.30	5	NO	NO	0
23	3000	3	complex	0.30	5	NO	NO	0
24	3000	4	complex	0.30	5	NO	NO	0

2.2 Results

We prepared the results in the same fashion for the extended simulation as we did for the simulation study in the manuscript, so that the results can be easily compared. We only evaluated the results for those conditions, where all 50 trials were completed, that is, only for conditions $1-8$, $11-17$, and 21.

Across conditions, we observed only a few numerical instabilities. For the unpenalized M2PCMPMs, the model failed to converge in 2% of trials (i.e., 1 trial) in only one condition (Condition 2: $L=4$, simple structure, $\rho=0$, $m=3$). For the lasso-penalized M2PCMPMs with orthogonal latent factor correlation matrix, all selected models (i.e., with the tuned hyperparameter η) converged. For the lasso-penalized M2PCMPMs with oblique latent factor correlation matrix, the selected model (i.e., with the tuned hyperparameter η) exhibited failed convergence in 2% of trials (i.e., 1 trial) in only one condition (Condition 8: $L=3$, complex structure, $\rho=0.3$, $m=3$). The results in the following have been computed under inclusion of all 50 trials in all fully completed conditions. This is more conservative, as parameter recovery estimates should be (if at all) negatively affected by the inclusion of the respective one trial with failed convergence.

The computation times are shown in Table 1.

Table 1: Average (mean) and median (med) computation times (in seconds) for the different models in the 16 conditions

Condition	Rotate		Lasso (ortho)		Lasso (obli)	
	mean	med	mean	med	mean	med
1: $L = 3$, simple, $\rho = 0$, $m = 3$, $N = 1200$	44800.30	37984.18	52708.97	52285.92	66467.76	65171.97
2: $L = 4$, simple, $\rho = 0$, $m = 3$, $N = 1200$	112319.66	102366.12	103742.49	88150.09	125510.26	119395.42
3: $L = 3$, complex, $\rho = 0$, $m = 3$, $N = 1200$	65001.83	62232.32	106045.49	103962.32	125019.00	123573.64
4: $L = 4$, complex, $\rho = 0$, $m = 3$, $N = 1200$	89772.61	72688.65	97680.21	93627.85	191025.80	168859.88
5: $L = 3$, simple, $\rho = 0.3$, $m = 3$, $N = 1200$	60612.08	58762.30	72128.73	70208.67	87336.47	84915.10
6: $L = 4$, simple, $\rho = 0.3$, $m = 3$, $N = 1200$	123243.72	103142.10	92826.15	85313.96	176297.66	157034.33
7: $L = 3$, complex, $\rho = 0.3$, $m = 3$, $N = 1200$	73348.88	70634.17	115713.70	107817.19	184646.78	176197.47
8: $L = 4$, complex, $\rho = 0.3$, $m = 3$, $N = 1200$	102121.53	82989.74	101243.87	84702.81	238410.55	222564.30
11: $L = 3$, complex, $\rho = 0$, $m = 5$, $N = 1200$	117642.22	105557.63	112059.23	104763.21	170163.51	154521.26
12: $L = 4$, complex, $\rho = 0$, $m = 5$, $N = 1200$	241491.82	212162.63	191052.40	176833.10	319658.77	306096.99
13: $L = 3$, simple, $\rho = 0.3$, $m = 5$, $N = 1200$	131809.66	120033.80	135454.31	134016.30	186029.34	166037.06
14: $L = 4$, simple, $\rho = 0.3$, $m = 5$, $N = 1200$	257943.53	210646.43	172928.85	172424.03	311803.17	264874.31
15: $L = 3$, complex, $\rho = 0.3$, $m = 5$, $N = 1200$	166777.70	134178.87	176866.40	157846.40	254077.42	229021.76
16: $L = 4$, complex, $\rho = 0.3$, $m = 5$, $N = 1200$	208420.50	186831.66	221388.70	203487.87	437641.73	436586.93
17: $L = 3$, simple, $\rho = 0$, $m = 5$, $N = 3000$	308047.51	286108.35	226350.01	187757.16	252632.75	236262.55
21: $L = 3$, simple, $\rho = 0.3$, $m = 5$, $N = 3000$	288808.23	240828.86	260568.05	246785.37	330098.50	309092.90

Notes. Note that the lasso models include hyperparameter tuning, and thus multiple model fits in one instance, but for the first hyperparameter grid value, the rotate fit parameter estimates were used as start values (yielding a 1-iteration run of the algorithm). obli = oblique (latent traits are a priori assumed to be correlated). ortho = orthogonal (latent traits are a priori assumed to be orthogonal). L = number of latent traits. ρ = true latent trait correlation. m = number of items per trait.

The average bias and RMSE across all items per condition are displayed in Table 2 for the δ_j parameters and in Table 3 for the $\log \nu_j$ parameters.

Table 2: Average bias (between-item SD in parentheses) and RMSE (between-item SD in parentheses) on δ_j parameters across all items per condition

Design					Bias (SD)			RMSE (SD)		
N	L	$\boldsymbol{\alpha}$ structure	ρ	m	Lasso (obli)	Lasso (ortho)	Rotate	Lasso (obli)	Lasso (ortho)	Rotate
1200	3	simple	0	3	0.003 (0.002)	0.003 (0.002)	-0.000 (0.001)	0.012 (0.002)	0.012 (0.002)	0.012 (0.002)
1200	3	simple	.3	3	0.003 (0.003)	0.001 (0.002)	0.000 (0.002)	0.012 (0.002)	0.011 (0.002)	0.011 (0.002)
1200	3	simple	.3	5	0.003 (0.002)	0.001 (0.002)	0.000 (0.002)	0.014 (0.003)	0.012 (0.003)	0.012 (0.003)
1200	3	complex	0	3	0.003 (0.002)	0.002 (0.001)	0.001 (0.001)	0.013 (0.003)	0.013 (0.002)	0.012 (0.002)
1200	3	complex	0	5	0.003 (0.001)	0.003 (0.002)	0.001 (0.001)	0.012 (0.004)	0.013 (0.004)	0.012 (0.004)
1200	3	complex	.3	3	0.001 (0.001)	-0.002 (0.001)	-0.002 (0.001)	0.014 (0.002)	0.013 (0.002)	0.013 (0.002)
1200	3	complex	.3	5	0.003 (0.001)	0.001 (0.001)	0.000 (0.001)	0.014 (0.003)	0.013 (0.004)	0.013 (0.004)
1200	4	simple	0	3	0.006 (0.004)	0.006 (0.004)	0.003 (0.003)	0.014 (0.004)	0.015 (0.004)	0.014 (0.003)
1200	4	simple	.3	3	0.006 (0.004)	0.002 (0.002)	0.001 (0.003)	0.014 (0.005)	0.013 (0.004)	0.013 (0.004)
1200	4	simple	.3	5	0.005 (0.002)	0.003 (0.001)	0.002 (0.001)	0.016 (0.003)	0.013 (0.003)	0.013 (0.003)
1200	4	complex	0	3	0.004 (0.002)	0.002 (0.003)	0.000 (0.003)	0.015 (0.004)	0.013 (0.004)	0.013 (0.004)
1200	4	complex	0	5	0.002 (0.002)	0.004 (0.002)	0.003 (0.002)	0.014 (0.003)	0.014 (0.003)	0.014 (0.003)
1200	4	complex	.3	3	0.006 (0.002)	0.002 (0.002)	0.001 (0.002)	0.017 (0.003)	0.014 (0.003)	0.014 (0.003)
1200	4	complex	.3	5	0.005 (0.003)	0.006 (0.002)	0.005 (0.002)	0.015 (0.003)	0.015 (0.003)	0.015 (0.003)
3000	3	simple	0	5	0.001 (0.001)	0.001 (0.001)	0.000 (0.001)	0.007 (0.002)	0.008 (0.002)	0.008 (0.002)
3000	3	simple	.3	5	0.001 (0.001)	0.000 (0.001)	-0.000 (0.001)	0.008 (0.002)	0.008 (0.002)	0.008 (0.002)

Notes. Note that rotated models have the same δ_j estimates regardless of rotation methods as those only affect $\hat{\boldsymbol{\alpha}}$. obli = oblique (latent traits are a priori assumed to be correlated). ortho = orthogonal (latent traits are a priori assumed to be orthogonal). L = number of latent traits. ρ = true latent trait correlation. m = number of items per trait.

Table 3: Average bias (SD in parentheses) and RMSE (SD in parentheses) on $\log \nu_j$ parameters across all items per condition

Design					Bias (SD)			RMSE (SD)		
N	L	$\boldsymbol{\alpha}$ structure	ρ	m	Lasso (obli)	Lasso (ortho)	Rotate	Lasso (obli)	Lasso (ortho)	Rotate
1200	3	simple	0	3	-0.008 (0.025)	-0.008 (0.027)	0.007 (0.021)	0.079 (0.023)	0.079 (0.024)	0.076 (0.022)
1200	3	simple	.3	3	-0.006 (0.013)	0.010 (0.016)	0.013 (0.017)	0.072 (0.023)	0.078 (0.026)	0.079 (0.026)
1200	3	simple	.3	5	-0.014 (0.015)	-0.004 (0.011)	-0.002 (0.011)	0.060 (0.017)	0.058 (0.015)	0.059 (0.016)
1200	3	complex	0	3	0.006 (0.015)	0.013 (0.016)	0.015 (0.016)	0.080 (0.026)	0.081 (0.028)	0.081 (0.028)
1200	3	complex	0	5	-0.004 (0.009)	-0.007 (0.022)	-0.003 (0.022)	0.057 (0.014)	0.059 (0.020)	0.058 (0.018)
1200	3	complex	.3	3	-0.007 (0.014)	0.007 (0.012)	0.009 (0.011)	0.075 (0.027)	0.075 (0.026)	0.075 (0.026)
1200	3	complex	.3	5	-0.014 (0.026)	-0.001 (0.013)	-0.000 (0.014)	0.062 (0.021)	0.059 (0.015)	0.059 (0.015)
1200	4	simple	0	3	-0.061 (0.121)	-0.089 (0.195)	-0.054 (0.148)	0.114 (0.107)	0.143 (0.173)	0.119 (0.129)
1200	4	simple	.3	3	-0.057 (0.109)	-0.041 (0.130)	-0.037 (0.127)	0.107 (0.096)	0.110 (0.119)	0.108 (0.115)
1200	4	simple	.3	5	-0.047 (0.068)	-0.031 (0.057)	-0.030 (0.058)	0.081 (0.060)	0.071 (0.047)	0.072 (0.047)
1200	4	complex	0	3	-0.042 (0.054)	-0.057 (0.180)	-0.049 (0.175)	0.094 (0.065)	0.118 (0.161)	0.116 (0.154)
1200	4	complex	0	5	-0.042 (0.071)	-0.042 (0.069)	-0.042 (0.073)	0.080 (0.061)	0.079 (0.058)	0.080 (0.062)
1200	4	complex	.3	3	-0.047 (0.095)	-0.044 (0.148)	-0.042 (0.148)	0.103 (0.088)	0.109 (0.128)	0.108 (0.127)
1200	4	complex	.3	5	-0.038 (0.058)	-0.030 (0.052)	-0.030 (0.053)	0.076 (0.049)	0.069 (0.040)	0.070 (0.041)
3000	3	simple	0	5	-0.004 (0.011)	-0.009 (0.028)	-0.007 (0.030)	0.036 (0.010)	0.040 (0.023)	0.041 (0.023)
3000	3	simple	.3	5	-0.012 (0.018)	-0.005 (0.014)	-0.004 (0.013)	0.040 (0.016)	0.038 (0.012)	0.038 (0.012)

Notes. Note that rotated models have the same δ_j estimates regardless of rotation methods as those only affect $\hat{\boldsymbol{\alpha}}$. obli = oblique (latent traits are a priori assumed to be correlated). ortho = orthogonal (latent traits are a priori assumed to be orthogonal). L = number of latent traits. ρ = true latent trait correlation. m = number of items per trait.

The bias and RMSE estimates across items for the multidimensional discriminations are shown in Figure 1. The estimates for the Mean Correct Estimation Rate (CER) for the different models is are shown in Figure 2. Condition average CER for the BIC-selected model (y-axis) are plotted against condition average CER for the CER-selected model (x-axis) in Figure 3.

We calculated the bias in the off-diagonal elements of $\hat{\boldsymbol{\alpha}} Cov(\boldsymbol{\theta}) \hat{\boldsymbol{\alpha}}^\top$. Table 4 and Figure 4 show that when factors are uncorrelated, Bias across all item pairs is mostly negligible. A latent correlation of 0.3 leads to some bias, but never larger than 0.02. RMSE also increases when the latent correlation is non-zero. The larger sample size of $N = 3000$ was no safeguard against Biases and RMSE consequently. Across conditions, ℓ_1-regularization had slightly lower RMSE and Bias. These findings are in line with the results of the simulation study reported in the main manuscript.

Figure 1: Distribution of bias (black) and RMSE (gray) estimates across items for each simulation condition. (Empty panels indicate that the corresponding condition was not completed within max wall time for all 50 trials; N = sample size. L = number of latent tarits. r = true correlation between latent traits. m = number of items per trait. simple / complex = type of $\boldsymbol{\alpha}$ structure. Lasso / Rotate = model variant. ortho = orthogonal. obli = oblique.)

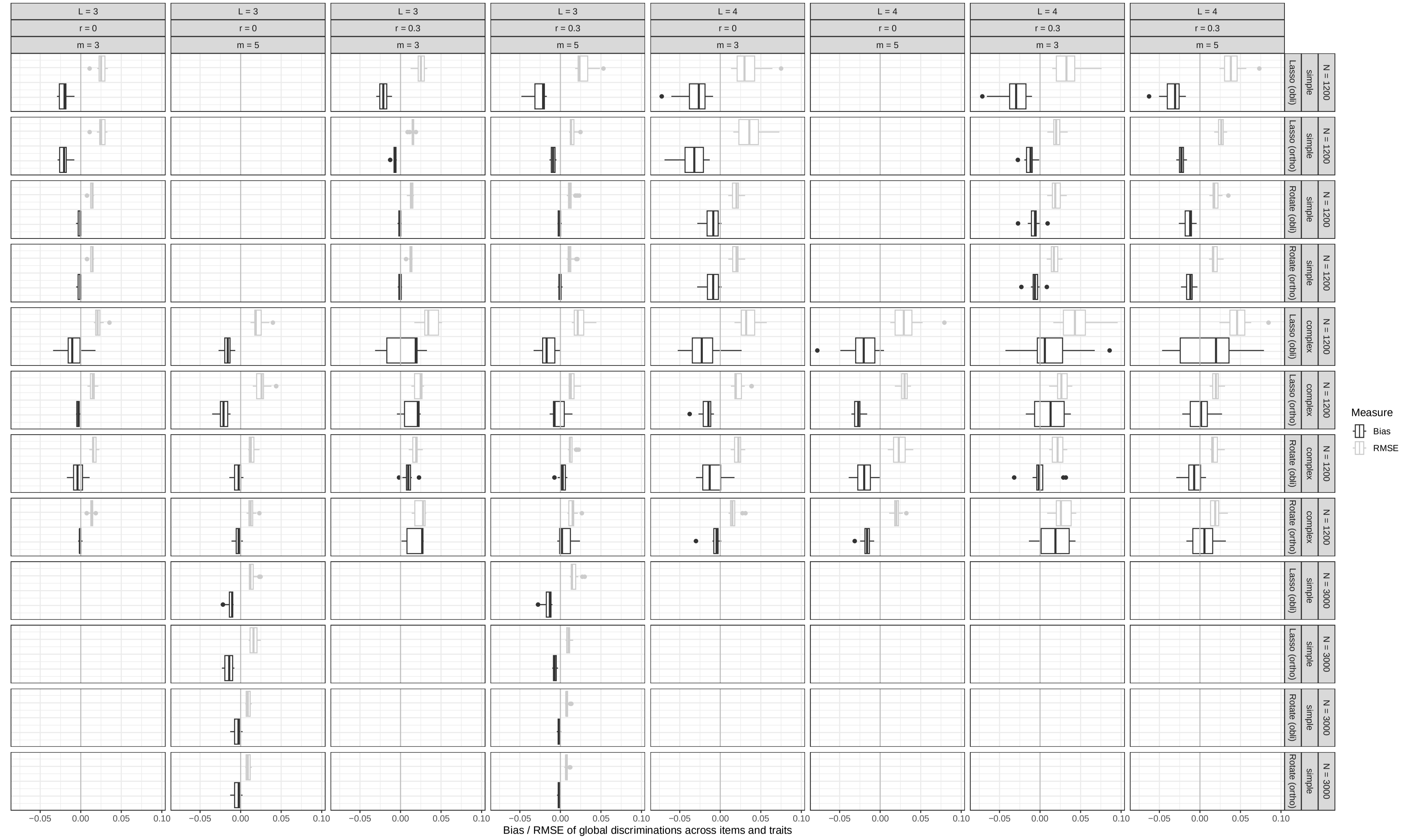

Figure 2: Mean Correct Estimation Rate (CER) estimates for each simulation condition. Estimates for the different model variants are shown on the x-axis and indicated by different shapes as detailed in the legend on the right-hand side. (Empty panels indicate that the corresponding condition was not completed within max wall time for all 50 trials; three rows for N = 3000 were omitted entirely as none of the conditions in them were completed in time; N = sample size. L = number of latent tarits. r = true correlation between latent traits. m = number of items per trait. simple / complex = type of $\boldsymbol{\alpha}$ structure. Lasso / Rotate = model variant. ortho = orthogonal. obli = oblique.)

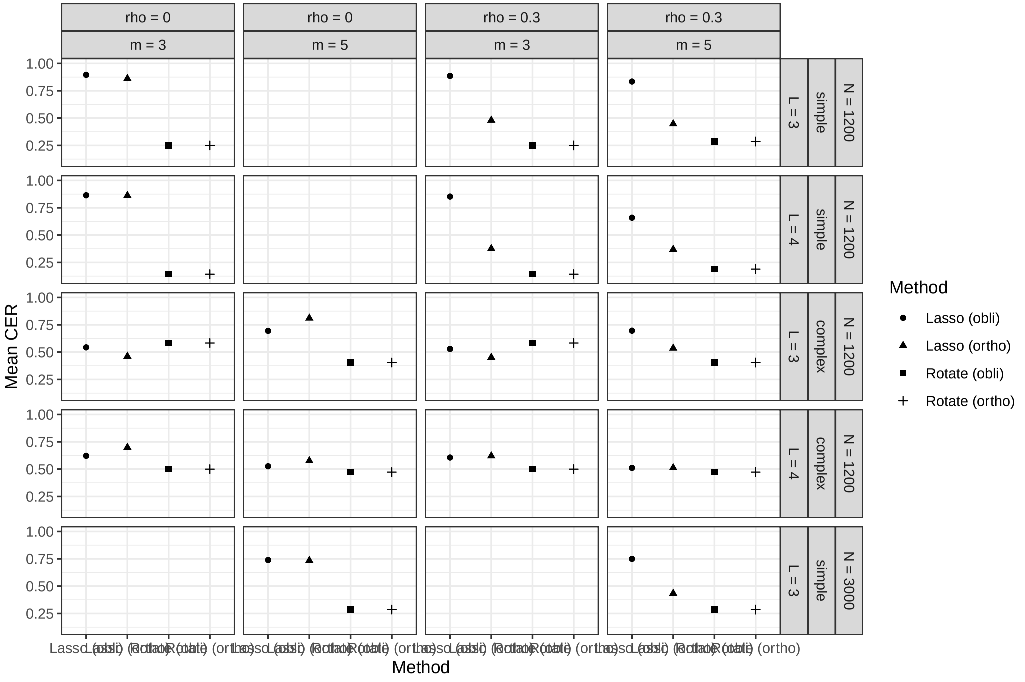

Figure 3: Condition average CER for the BIC-selected model (y-axis) against condition average CER for the CER-selected model (x-axis), shown in two separate panels (lasso with oblique latent covariance matrix on the left and lasso with orthogonal latent covariance matrix on the right). Simulation conditions (only fully completed conditions are shown; in terms of sample size (N), number of latent traits (L), latent factor correlation (r), and number of items per trait (m)) are shown in different colours as indicated by the legend on the right-hand side (under "Condition"). Different $\boldsymbol{\alpha}$ structures are represented by different shapes as indicated by the legend on the right-hand side (under "Structure").

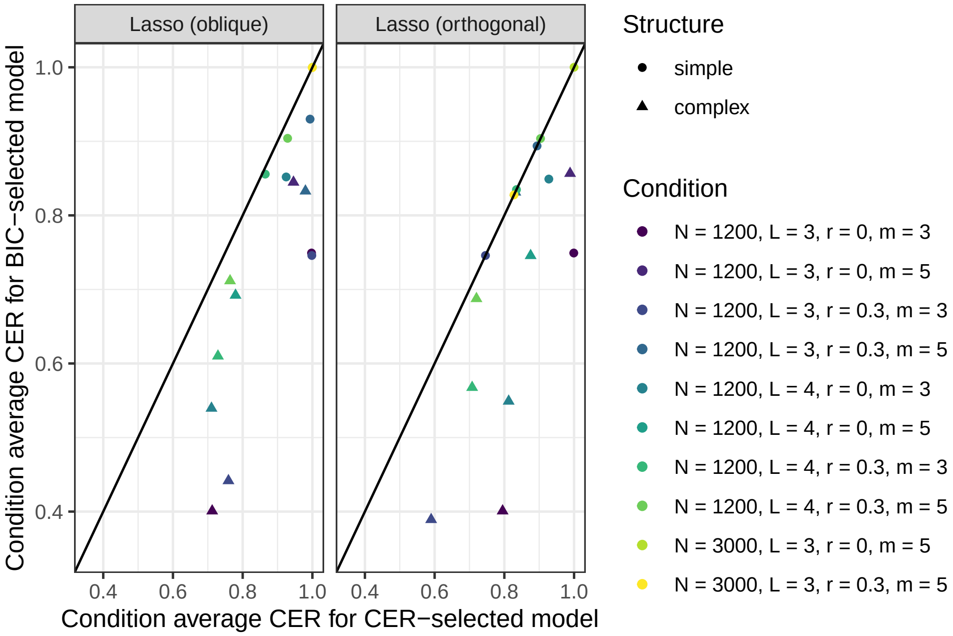

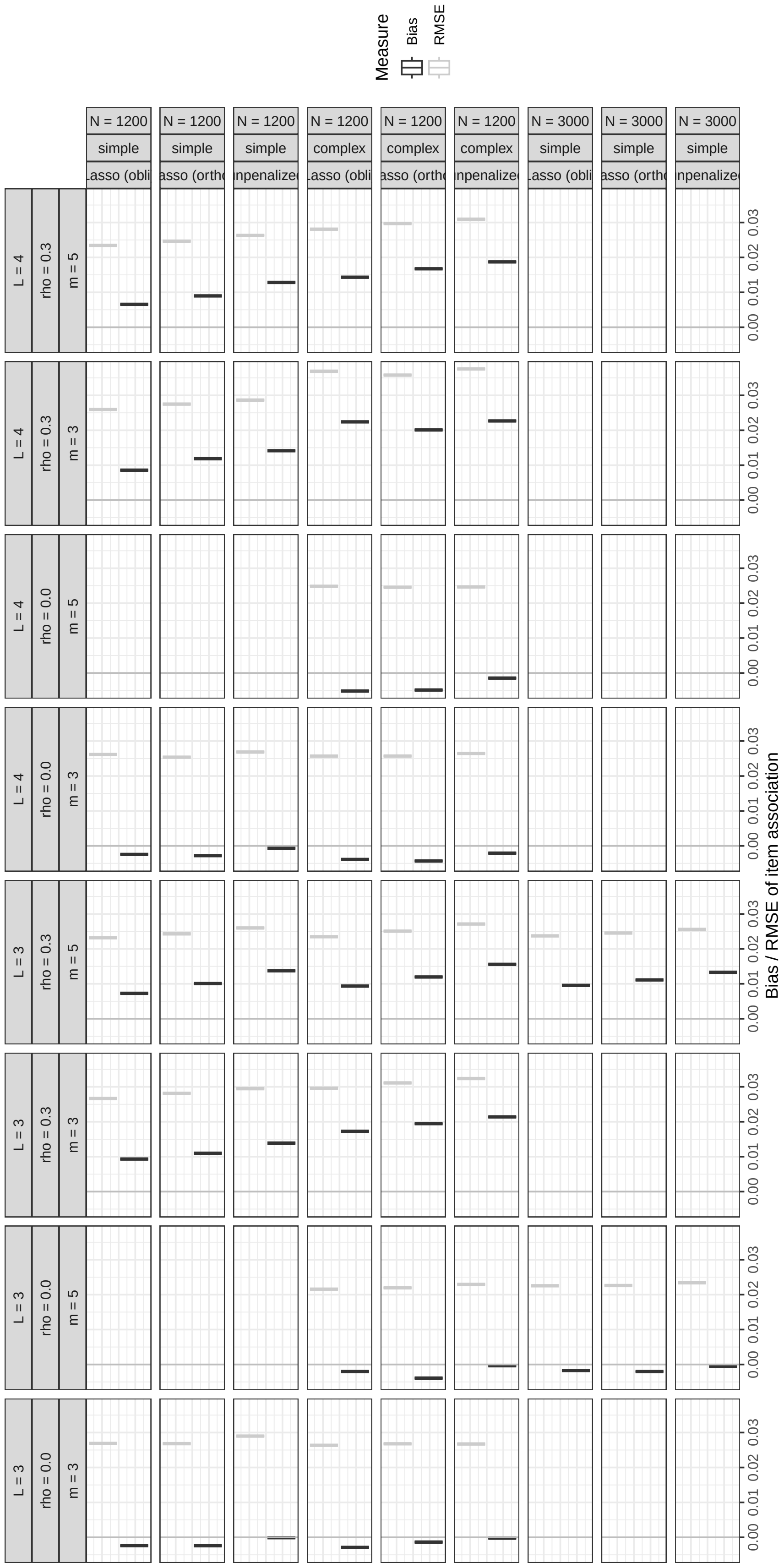

Figure 4: Bias and RMSE of the off-diagonal elements of $\hat{\boldsymbol{\alpha}}Cov(\boldsymbol{\theta})\hat{\boldsymbol{\alpha}}^{\top}$, a measure of association of item pairs.

Table 4: Bias and RMSE in the off-diagonal elements of $\hat{\boldsymbol{\alpha}} Cov(\boldsymbol{\theta}) \hat{\boldsymbol{\alpha}}^\top$.

Design					Bias			RMSE		
N	L	$\boldsymbol{\alpha}$ structure	ρ	m	ℓ_1 (obli)	ℓ_1 (ortho)	unpen.	ℓ_1 (obli)	ℓ_1 (ortho)	unpen.
1200	3	simple	0.0	3	-0.000	-0.002	-0.002	0.029	0.027	0.027
1200	3	complex	0.0	3	-0.000	-0.001	-0.003	0.027	0.027	0.026
1200	4	simple	0.0	3	-0.001	-0.003	-0.002	0.027	0.025	0.026
1200	4	complex	0.0	3	-0.002	-0.004	-0.004	0.026	0.026	0.026
1200	3	simple	0.3	3	0.014	0.011	0.009	0.029	0.028	0.027
1200	3	complex	0.3	3	0.021	0.019	0.017	0.032	0.031	0.030
1200	4	simple	0.3	3	0.014	0.012	0.009	0.029	0.027	0.026
1200	4	complex	0.3	3	0.023	0.020	0.022	0.038	0.036	0.037
1200	3	complex	0.0	5	-0.000	-0.004	-0.002	0.023	0.022	0.022
1200	4	complex	0.0	5	-0.001	-0.005	-0.005	0.025	0.025	0.025
1200	3	simple	0.3	5	0.014	0.010	0.007	0.026	0.024	0.023
1200	3	complex	0.3	5	0.016	0.012	0.009	0.027	0.025	0.023
1200	4	simple	0.3	5	0.013	0.009	0.007	0.026	0.025	0.023
1200	4	complex	0.3	5	0.019	0.017	0.014	0.031	0.030	0.028
3000	3	simple	0.0	5	-0.001	-0.002	-0.002	0.023	0.023	0.023
3000	3	simple	0.3	5	0.013	0.011	0.010	0.026	0.025	0.024

References

Microsoft Corporation, & Weston, S. (2022). doparallel: Foreach parallel adaptor for the 'parallel' package [Computer software manual]. Retrieved from `https://CRAN.R-project.org/package=doParallel` (R package version 1.0.17)
